# Assessing the format and content of journal published and non-journal published rapid review reports: A comparative study

**DOI:** 10.1371/journal.pone.0238025

**Published:** 2020-08-26

**Authors:** Chantelle Garritty, Mona Hersi, Candyce Hamel, Adrienne Stevens, Zarah Monfaredi, Claire Butler, Andrea C. Tricco, Lisa Hartling, Lesley A. Stewart, Vivian Welch, Kednapa Thavorn, Wei Cheng, David Moher

**Affiliations:** 1 Clinical Epidemiology Program, Ottawa Hospital Research Institute, Ottawa, Canada; 2 TRIBE Graduate Program, University of Split School of Medicine, Split, Croatia; 3 Li Ka Shing Knowledge Institute, St. Michael's Hospital, Toronto, Canada; 4 Dalla Lana School of Public Health, University of Toronto, Toronto, Canada; 5 Alberta Research Centre for Health Evidence, Department of Pediatrics, University of Alberta, Edmonton, Canada; 6 Centre for Reviews and Dissemination, University of York, York, United Kingdom; 7 Methods Centre, Bruyère Research Institute, Ottawa, Canada; 8 School of Epidemiology and Public Health, University of Ottawa, Ottawa, Canada; Universitat de Valencia, SPAIN

## Abstract

**Background:**

As production of rapid reviews (RRs) increases in healthcare, knowing how to efficiently convey RR evidence to various end-users is important given they are often intended to directly inform decision-making. Little is known about how often RRs are produced in the published or unpublished domains, and what and how information is structured.

**Objectives:**

To compare and contrast report format and content features of journal-published (JP) and non-journal published (NJP) RRs.

**Methods:**

JP RRs were identified from key databases, and NJP RRs were identified from a grey literature search of 148 RR producing organizations and were sampled proportionate to cluster size by organization and product type to match the JP RR group. We extracted and formally compared ‘how’ (i.e., visual arrangement) and ‘what’ information was presented.

**Results:**

We identified 103 RRs (52 JP and 51 NJP) from 2016. A higher percentage of certain features were observed in JP RRs compared to NJP RRs (e.g., reporting authors; use of a traditional journal article structure; section headers including abstract, methods, discussion, conclusions, acknowledgments, conflict of interests, and author contributions; and use of figures (e.g., Study Flow Diagram) in the main document). For NJP RRs, a higher percentage of features were observed (e.g., use non-traditional report structures; bannering of executive summary sections and appendices; use of typographic cues; and including outcome tables). NJP RRs were more than double in length versus JP RRs. Including key messages was uncommon in both groups.

**Conclusions:**

This comparative study highlights differences between JP and NJP RRs. Both groups may benefit from better use of plain language, and more clear and concise design. Alternative innovative formats and end-user preferences for content and layout should be studied further with thought given to other considerations to ensure better packaging of RR results to facilitate uptake into policy and practice.

**Study registration:**

The full protocol is available at: https://osf.io/29xvk/.

## Introduction

There are many obstacles to the use and uptake of systematic reviews (SRs) that render most underutilized [1–4]. A significant barrier is that SRs can be difficult and time-consuming to conduct, usually taking 1 to 2 years to complete [5, 6]. They can also be lengthy to read, especially to those who seek information in a convenient, portable, and timely manner. Format and content features of SRs have been identified among the main barriers to their uptake by policymakers and healthcare managers [7]. Studies that have examined tailoring of SR content and format for end-users (i.e., clinicians, health policymakers, and health system managers) [8–14], suggest that users favour clear, concise summaries in simple, easy to understand language [9, 11–14]. Further, evidence summaries of SRs are likely more straightforward to understand than complete SRs [14]

Rapid reviews (RRs) have emerged as a form of knowledge synthesis that shortens or omit components of the SR process to produce information in a timelier manner than most SRs [5, 15–17]. Researchers often tailor the methods used in RRs according to the knowledge user request, available budget, and timeline of usually a few weeks to six months (S1 File) [18]. Several organizations have undertaken RRs using various approaches in their conduct [19–21], and they have become a valuable information tool to support the use of evidence for decision-making [22]. Yet, we know little about what and how information is conveyed in RRs or the extent to which tailored formats are used beyond the conventional IMRaD (introduction, methods, results and discussion) structure widely used by journals across many disciplines, including healthcare. IMRaD is the standard format of academic biomedical journal articles, including published SRs [23] and is explicitly recommended by the International Committee of Medical Journal Editors (ICMJE) [24].

Although health research is often conveyed to decision-makers using the IMRaD format, some suggest this format may hinder use for decision-making purposes by clinicians, policymakers and other stakeholders [25]. In contrast to this, others have developed alternative formats; namely, those described as ‘graded entry’ involving material organized to highlight decision-relevant, summarized information upfront with access to more detailed information gradually uncovered for the reader [1, 11, 26, 27] (S2 File). For these products, the fixed IMRaD structure has been set aside and instead, key information is arranged to facilitate scanning of the most relevant information upfront.

As the production of RRs grows, it is increasingly vital that we understand the most effective and efficient ways to deliver RR evidence to various end-users. Ideally, RR producers should be guided by elements of good document design, including ‘how’ best to layout information and ‘what’ information or content is of most use and value to include. Given what we know about the challenges SRs have faced regarding adequate content and format [1, 7, 11, 28, 29], RRs may, too, be prone to some of these same obstacles. However, to date, only indirect research exists from SRs, as no studies have carefully examined this issue for RRs. Therefore, the main objective of this study was to determine the format and content of RRs based on the systematic identification of an international sample of both journal-published (JP) and non-journal-published (NJP) RRs and to compare and contrast features between them. We chose this comparison to reflect real-world use of RRs, as we know that several organizations around the globe are producing them but are not necessarily publishing them in journals. By eliciting this information, we aim to establish a baseline of data on the production and design of RRs and to highlight future considerations to enhance features leading to better use and uptake in decision-making.

## Methods

Below is an abridged version of the methods. Full methods details are provided elsewhere (S3 File).

### Study design

We conducted a descriptive, comparative study of a broad selection of RRs. All variables and analyses were determined a priori as per the protocol (https://osf.io/29xvk/).

### Defining ‘format’ and ‘content’

We defined format or layout to mean ‘how’ information was presented (i.e., the visual arrangement, appearance, or presentation of information contained within a report) with content referring to the main features of a report in terms of ‘what’ information was presented (e.g., included sections or information).

### Search strategy and process

#### Bibliographic searching to identify journal published (JP) RRs

We developed a draft bibliographic database search strategy for MEDLINE (CG and AS) vis-à-vis key ‘seed’ articles. This was peer-reviewed by a senior information specialist (BS) using the PRESS checklist [30]. We then modified final MEDLINE search for eight other bibliographic databases (S4 File). We did not apply language restrictions but restricted reports to those published in 2016.

#### Grey literature searching to identify non-journal published (NJP) RRs

We searched websites listed in CADTH’s Grey Matters checklist [31] and the PROSPERO register. Further, we searched the websites and a contact list of pre-identified organizations (n = 148) that produce or commission RRs. If a RR did not report methodology or the reported methodology was unclear, we contacted authors for further information. As a proxy, we used any available internal methods guidance documents as requested and provided by authors/organizations.

#### Non-journal published (NJP) RRs sampling strategy

We identified a mix of higher and lower RR volume-producing organizations through grey literature searching efforts. Since a large number of identified RRs were likely to be clustered by organization, we first catalogued the retrieved sample of NJP RRs by organization and then by product per organization. Next, we identified the total number of clusters from across all of the organizations and sampled RRs from each proportionate to cluster size. In some cases, this meant that sampling took place at the organizational level and by RR type within an organization. For the sake of feasibility, we used the sample size of the JP group to determine the sample size in the NJP group.

### Sample size

We did not calculate a sample size for this descriptive study. However, we limited our sample for the sake of practicality using the abovementioned sampling strategy and to ensure comparison groups of similar sizes.

### Study selection

First, we applied eligibility criteria (S5 File) to screen bibliographic results from the journal published domain. One person reviewed the titles and abstracts while a second person reviewed the excluded citations. Two people independently reviewed full-text reports with disagreements resolved by consensus or a third person. We pilot tested a selection of records for title/abstract and full-text screening. Based on the screening of the JP group, we determined the number of RRs from the grey literature results needed to create a similar sample size in the NJP RR group. After sampling, the NJP group underwent the same screening process. We outlined the reasons for exclusion in a study flow diagram.

### Data collection

We extracted information specific to features of the reports across four broad categories considered to be involved in good document design, and that was most relevant given the nature of our study [32]. These included: 1) *report identifying information*; 2) *structure* (document organization); 3) *content*; 4) *visual design* covering legibility, graphic elements, and general layout. We also collected information on *other factors*, including the placement of certain sections in the report, how the report format was decided, and whether stakeholders provided input on the layout (S6 File). We piloted forms using a subset of articles. For general characteristics, one individual extracted data, while a second person verified a minimum 10% random sample of studies. We did full verification for all format outcomes.

We also assessed the *readability* (or the ease with which the reader can understand the written text) of the abstract, introduction, and discussion sections of the RRs using the Simple Measure of Gobbledygook (SMOG) readability test [33], used in studies assessing health information [34]. An online calculator provided scores corresponding to the level of education required to understand the analyzed text. We used Microsoft Word to give the *word count* of the main body of the report (i.e., all sections excluding references and appendices) and the total length of the document.

Given the rise of illegitimate publishing entities, we confirmed peer-review by first cross-checking each journal against the Directory of Open Access Journals (DOAJ) and assessing each journal according to a list of salient characteristics of predatory journals [35]. For NJP RRs, we noted if peer review was reported in the citation or if methods guidance or website information indicated peer review was part of their RR process.

### Data analysis

We reported the study characteristics of the RRs in tables and figures. For the main comparison (i.e., JP vs. NJP), we summarized characteristics using frequencies and/or proportions accompanied by appropriate statistical tests to determine if significant differences existed across variables between these groups concerning their journal or non-publication status. The estimated associations were crude and based on univariate analysis and, therefore were not adjusted for other factors. For a subset of features, we only reported numerical differences between the JP and NJP RRs, given any differences noted would likely be due to the distinct nature of biomedical journal publishing versus the in-house publishing structures of most healthcare research organizations producing RRs. Therefore, we only applied formal testing to a select group of variables where appropriate using a significance level of 0.05. Planned subgroup analyses (i.e., according to report structure, report production, the purpose of the RR, timeframe of conduct, peer review status, and funding sources) were not possible due to insufficient data.

To the extent possible, we followed the STROBE Statement—Checklist for cross-sectional studies as a proxy as no reporting guidance exists for this type of methodological research.

## Results

### Search results

**Fig 1:**
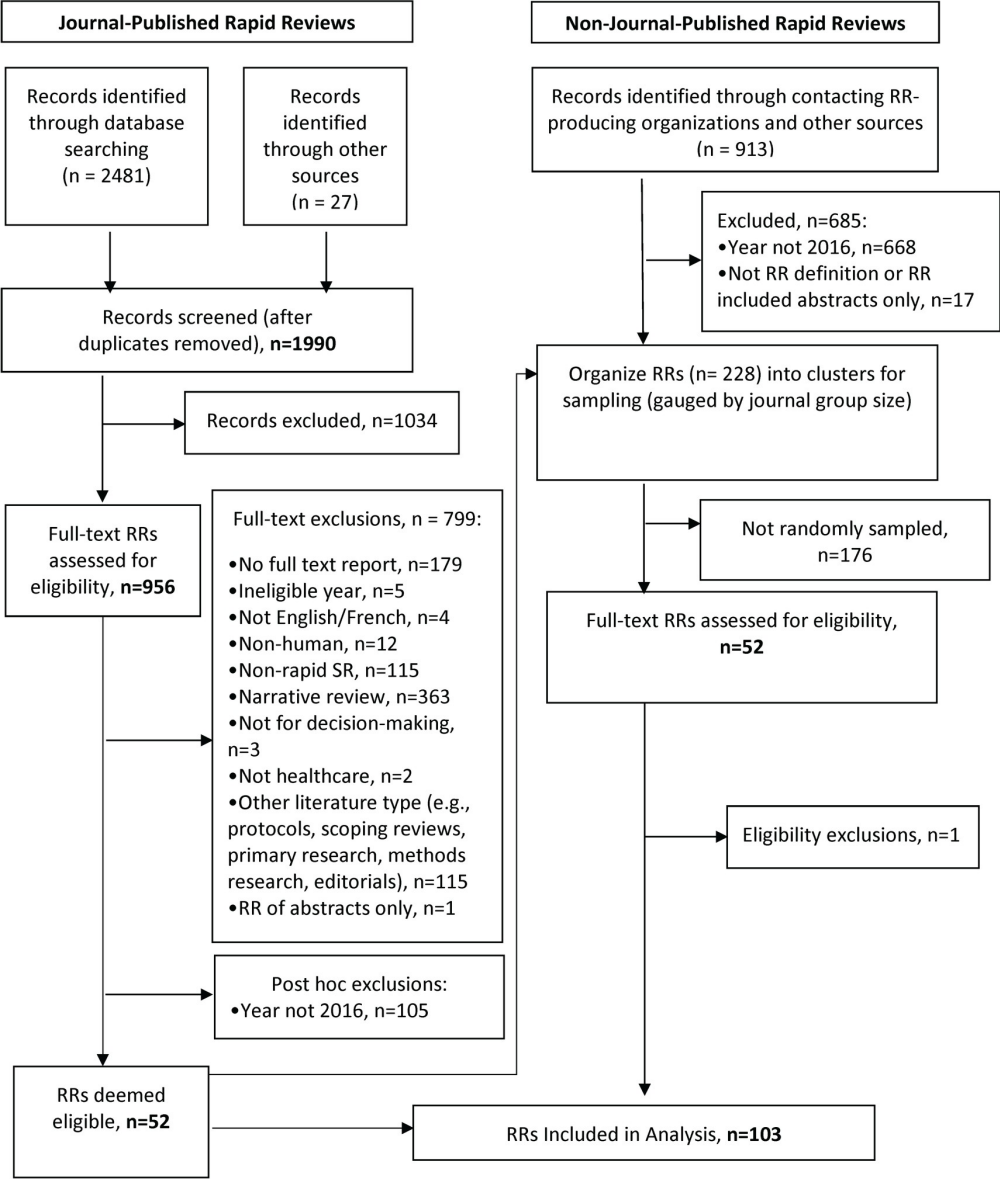
Study flow diagram. Breakdown of the number of rapid review reports identified, assessed for eligibly, and finally included in the main sample.

There were 2,508 records identified by the search for published RRs. After removing duplicates, there were 1,990 titles and abstracts screened that led to the exclusion of 1,034 records. Of the 956 full text articles retrieved, 52 JP RRs were eligible for inclusion. We identified NJP RRs by contacting RR producing organizations that resulted in 228 full-text reports; we organized these into clusters, which after sampling, resulted in 51 eligible full-text RR reports. In total, 103 RRs were included for analysis, as outlined in the study flow diagram (Fig 1).

### Characteristics of the identified RRs

General study characteristics and specific features of the included RRs reports are reported elsewhere (Tables 1 and 2 in S1 Table). JP RRs were published in 47 unique journals, all deemed legitimate (S2 Table). NJP RRs were identified from 25 individual organizations (S7 File). Substantial differences between JP and NJP RRs were noted, for example, for reporting the corresponding author (88% vs 6%), reporting of funding (75% vs 55%), and if the RR had undergone peer review (96% vs 12%). However, more NJP RRs were, for example, requested or commissioned (53% vs 25%) and were publicly available compared to RRs published in open access journals free of charge (98% vs 69%). Only one NJP RR was in French; all other RRs were in English.

A purpose or rationale for undertaking a RR was similarly reported across both groups (JP, 63% vs NJP, 59%). Only three (6%) RRs in each group indicated the time it took to produce the review, which ranged between 8–32 weeks for the JP RRs and 4–17 weeks for the NJP RRs. More NJP RRs reported end-user consultations during the development of the RR compared to JP RRs (57% vs 35%).

### Comparison of layout and content between published and non-journal published RRs

We present only notable findings in detail. For full results see Table 3 in S1 Table.

#### Report identifying information

*Authorship Reported*. All JP RRs (100%) reported the authors compared to NJP RRs (73%; p<0. 0001). For JP RRs, authorship was primarily cited in the byline of the article following the title (83%); authorship was rarely included here for NJP RRs (6%), with most (42%) listed in other places throughout the document (e.g., in the header).

#### Structure (document organization)

**Fig 2:**
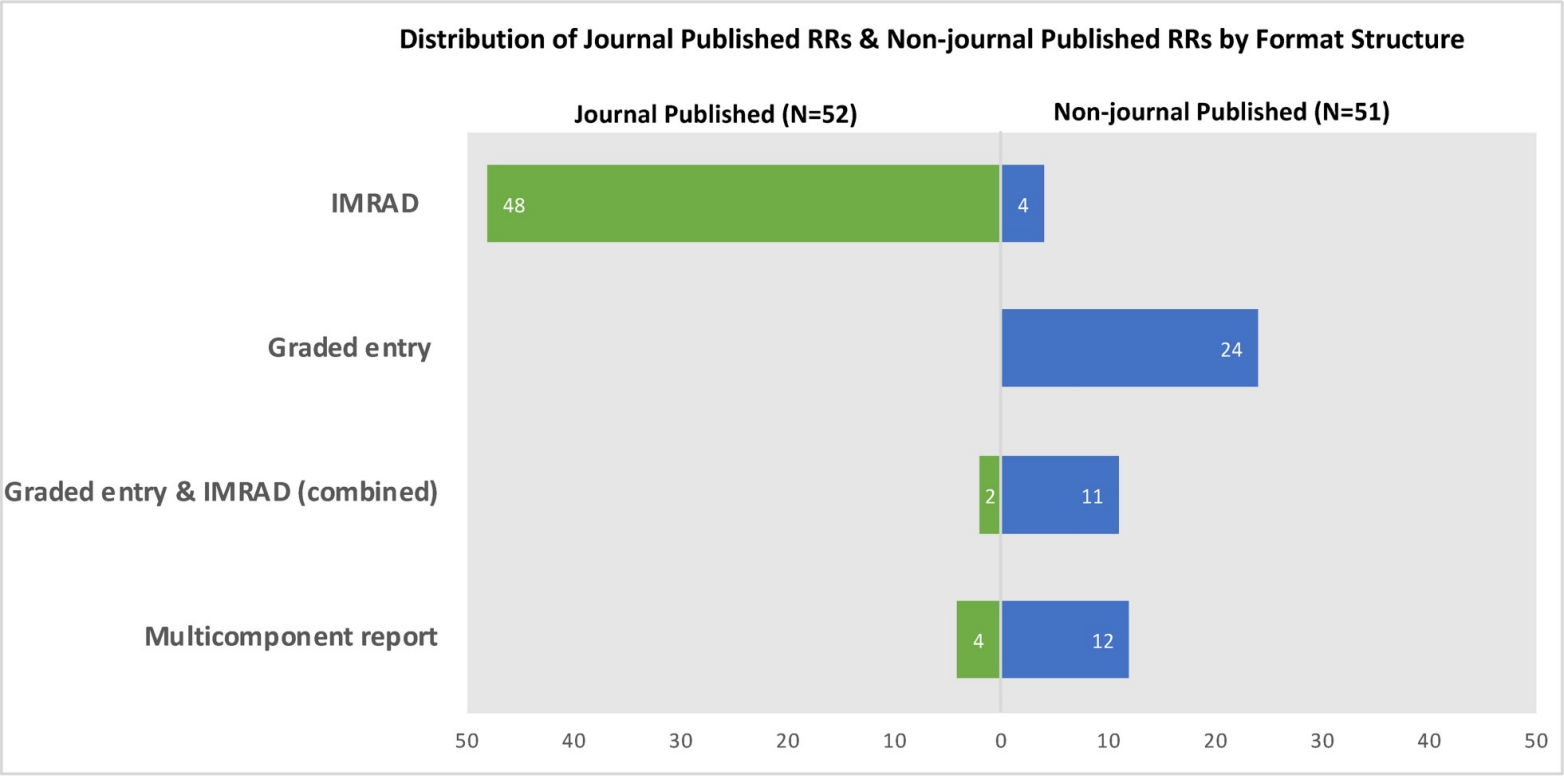
Rapid review format structures identified. Breakdown and comparison of the types of different rapid review report format structures identified across the journal published and non-journal published groups.

**Fig 3:**
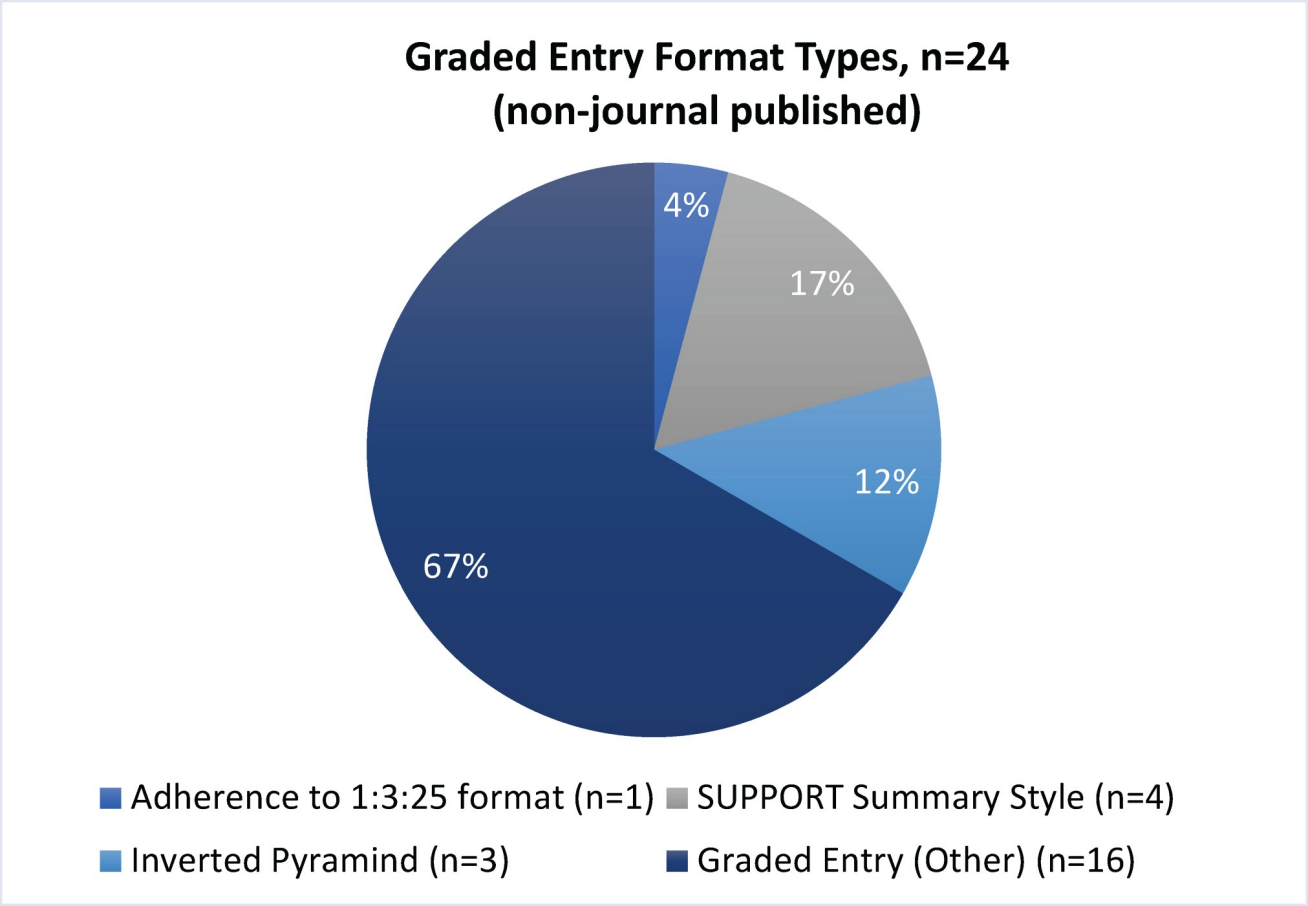
Graded entry formats identified. Breakdown of the subtypes of graded entry formats identified from the non-journal published rapid review reports.

*Type of report structure*. As typical with journal publications, a higher proportion of JP RRs was constructed according to the traditional IMRaD format when compared to NJP RRs [92% vs 8%; OR 125.49, 95% CI: 28.75–792.06]. Instead, almost half of NJP RRs (47%) were organized using a graded entry format, while no JP RRs used this structure (Fig 2). Graded entry front end combined with an IMRaD structured report was more common in NJP RRs than JP RRs, 22% vs 4%, respectively (Fig 2). We deemed nearly one-quarter of the NJP RRs (24%) to be multicomponent reports while few JP RRs used this format (4%) (Fig 2). The multicomponent report format type was added during the conduct of the study to capture those reports that were comprised of various components divided into lengthier 'chapters' or 'sections' beyond typical sections found in either the IMRAD or main graded entry structures. Additional chapters or sections of these reports included, for example, recommendations to guide policy and practice; health coverage information; and comparative information from other jurisdictions. Among NJP, the most common type of graded-entry report was a mix of graded entry styles within the same report structure (n = 16) (Fig 3). These reports did not ascribe to any of the other graded entry formats but did aim to highlight conclusions or key findings upfront followed by other report components that provided additional details. For example, some reports started with key messages, a brief description of methods in call-out boxes, and a summary of findings in a table, with additional information provided in appendices. Among other examples, there were reports that provided context and key points on the first page, with a synopsis of the methods that appeared before the introduction, or those that first provided a short summary of the methodological approach taken, the scope of the review, followed by a two-page evidence summary, and included additional abridged sections outlining the background, aims, and an overview of the evidence informing the review, ending with a section on the RR methods.

*Page numbering in the document and page length*. All RRs, except for three NJP RRs, had page numbering. Overall, JP RRs were considerably shorter than NJP RRs in page length of the main report [JP Mean (SD) 12.17(10.40); NJP Mean (SD) 27.14(25.22)], as well as for the complete report and the executive summary (Table 3 in S1 Table).

#### Content

**Fig 4:**
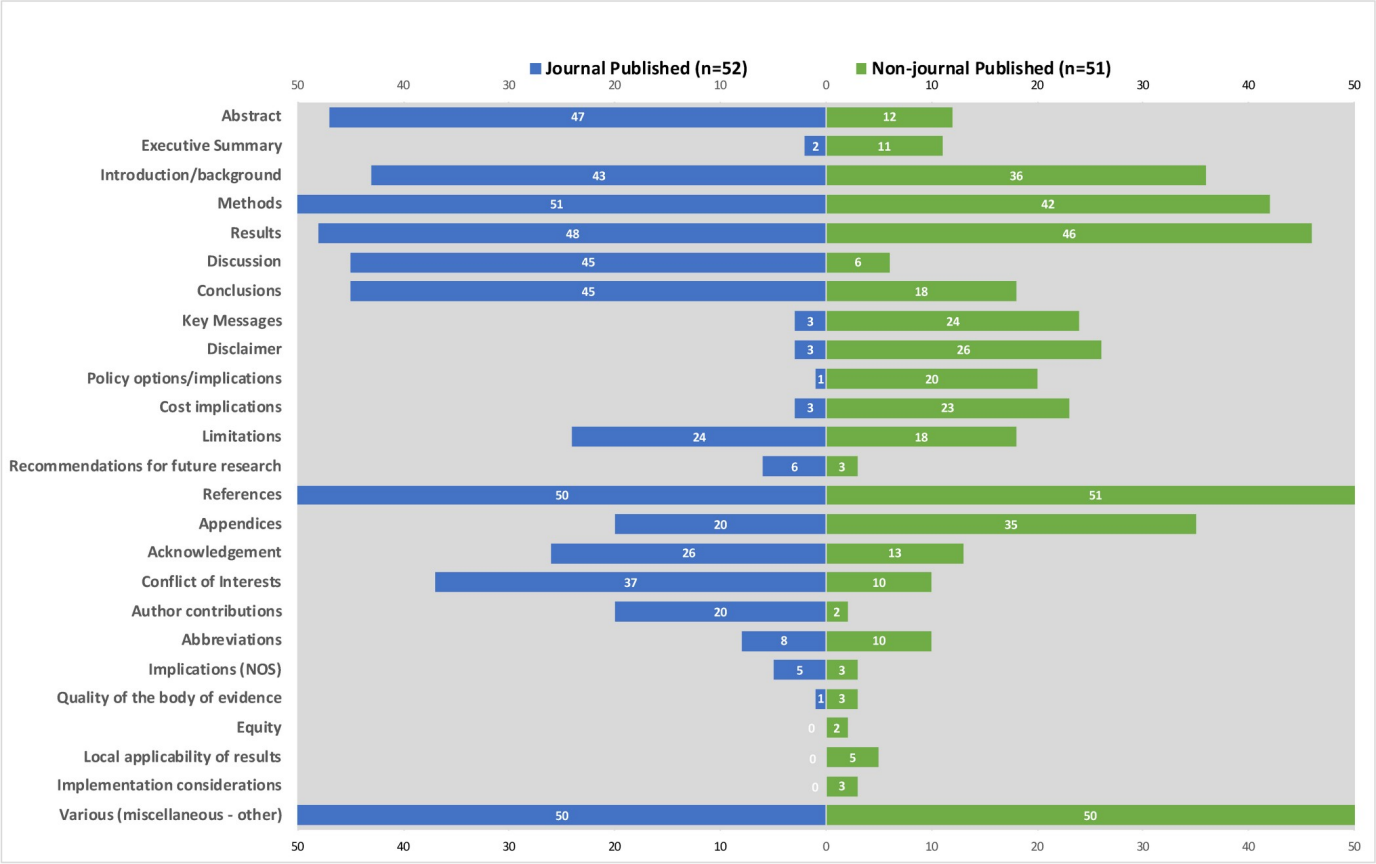
Bannering of content in rapid review reports. Breakdown and comparison of labelled sections identified across the journal published and non-journal published groups.

*Included banners and headers*. When we examined the components of the individual reports (Fig 4), we found a higher number of sections labelled across JP RRs when compared to the NJP RR reports. Sections included the following: abstracts; methods; discussion; conclusions; acknowledgements; conflicts of interest; and author contributions (See Table 3 in S1 Table for corresponding ORs, 95% CIs, and p-values). However, we found that JP RRs were less likely to include sections bannered as executive summary; key messages; disclaimer; policy options or implications; cost implications; and appendices. We did not find any notable differences for other bannered sections, including introduction or background, results, limitations, recommendations for future research, references or abbreviations. Few RRs from either group included an implications section or reported on the quality of the body of evidence. Only the NJP RRs included bannered sections on equity (n = 2), local applicability of results (n = 5), and implementation considerations (n = 3). Of the labels we identified, some of them potentially overlap and could refer to similar concepts (e.g., recommendations for future research, implications, and implementation). However, in this study, we did not formally assess the specific content of the bannered sections.

### Visual design

#### Legibility

*Document preparation system and typeface*. When examining components of *legibility*, or the ease with which a reader can recognize individual characters in the text, we judged the majority of the JP RRs to have been prepared using a professional publishing platform (92%). However, four JP RRs appear to have been prepared for publication using a desktop publishing software. We determined that most NJP RRs (76%) were likely developed using a desktop publishing software or produced in Microsoft Word and then converted to a portable document file (PDF) to be made publicly available online. When we assessed typeface, more JP RRs were prepared using a serif font for the *main text* when compared to NJP RRs [85% vs 25%; OR 15.51, 95% CI: 5.51–48.98)] that more often used a san-serif font. The typeface of the *headers* in the main text was predominantly serif for the JP RRs (69%) and sans-serif typeface for NJP RRs (86%).

#### Graphic elements

*Use of typographic cues and main document text*. When examining use of *typographic cues* in the RRs, fewer JP RRs used bolded text, keywords or phrases [10% vs 33%; OR 0.22, 95% CI: 0.06–0.69], underlining of text, keywords or phrases [2% vs 57%; OR 0.02, 95%: 0.00–0.12], and the use of bullet lists [48% vs 86%; OR 0.15, 95% CI: 0.05–0.42)]. We did not find any other variances in the use of bolded headers, use of colour to highlight text, keywords or phrases, call-out boxes, and use of italics to highlight text, keywords or phrases. For both JP and NJP RRs, the main body of the reports were mainly presented in monochrome (black, white or greyscale) (JP, 75% vs. NJP, 71%). Of the RRs that employed colour, all used a white background, with text black or dark blue, with various accent colours (e.g., blue, green).

*Tables in the main document and tables types*. Most RRs presented tables in the main body of the RR (JP, 87% vs. NJP, 88%), with a median (range; IQR) of 2 (1–17; 3) for JP RRs and 6 (1–33; 8.75) for NJP RRs. JP RRs were less likely to include outcome-specific data tables when compared to NJP RRs [6 vs 18; OR 0.24, 95% CI: 0.07–0.72]. Other types of tables included characteristics of included studies, general summary of findings tables, and quality assessment tables (Table 3 in S1 Table). Only one JP and one NJP RR included GRADE Summary of Findings tables in the main report.

*Materials provided in appendices*. Fewer JP RRs provided materials in the appendices when compared to NJP RRs [52% vs 73%; OR 0.41, 95% CI: 0.17–1.00]. Table 3 in S1 Table provides a list of the types of content provided in the appendices (e.g., search strategies, evidence tables).

*Figures in the main document*, *figure types*, *and figures in the appendices*. A greater proportion of JP RRs included figures in the main body of the RRs when compared to NJP RRs [73% vs 49%; OR 2.79, 95% CI: 1.15–7.01] with a median (range; IQR) of 1 (1–8; 1) for the JP RRs and 2 (1–11; 3) for the NJP RRs. However, JP RRs were more likely to include a PRISMA flow diagram (n = 34) versus NJP RRs (n = 12) [OR 6.02, 95% CI: 2.40–16.03]. Other types of drawings or schematics (e.g., analytic framework) were often included (JP = 15; NJP = 22). Only one RR from each group included Forest plots, while none included funnel plots. JP RRs were less likely to include figures in the appendices when compared to NJP RRs [4% vs 41%; OR 0.06, 95% CI: 0.01–0.27]. For many NJP RRs, we identified the PRISMA flow diagrams (15/21) in the appendices.

#### Other factors related to layout

*Placement of the methods section*, *key messages*, *and disclaimer*. All but one JP RR included the ‘methods section’ at the front end of the report, while only half of NJP RRs of the 42 RRs that had a labelled methods section did [98% vs 50%; OR 48.94, 95% CI: 7.01–2123.17]. The rest of the NJP RRs placed the methods section in either at the back end (n = 4) of the main report or in the appendices (n = 17). Only three JP RRs contained key messages compared to 24 NJP RRs. Similarly, three JP RRs included a disclaimer, while 26 NJP RRs provided this. We found key messages and disclaimers commonly reported at the front end of the report for both review types.

*Determination of the final report format*, *stakeholder input*, *availability of additional materials*. The final report layout for JP RRs was determined by the journals in which they were published. However, the majority of NJP RRs (94%) did not report how the final format was established, with only one report determined by the producer and two reports decided by the requestor/commissioner. Moreover, none of the NJP RRs reported if stakeholders had any input with regards to the final layout of the end-product. Few RRs indicated that additional material was available upon request (JP, 4% vs. NJP, 6%).

#### Readability

*SMOG index and word count*. According to the SMOG formula, there were no differences in the readability scores of JP RRs and NJP RRs in the abstract/summary, introduction/background, or discussion/conclusions sections. Across the RRs, SMOG scores indicated that between 13. 57–14.35 years of education would be needed to understand the writing contained in these selected sections of the RRs. JP RRs had significantly fewer words than NJP RRs in both the main body of the text [MD (SE): -3,561 (1,388), p = 0.01] and the entire document MD (SE): -7,050 (2,566); p = 0.01)].

## Discussion

This study systematically identified a diverse sample of RRs and discovered some similarities as well as differences between the published and unpublished RRs. At the outset, we understood that the nature of biomedical journal publishing would drive specific differences between groups and the fact that journals regulate the presentation of findings in the papers they publish. Similarly, we anticipated that NJP RRs would likely differ from JP RRs, given the specific mandates of healthcare organizations and the degree of independence to design and develop RR products for various knowledge-user audiences. Our results did reflect particular distinctions in format and content.

*Report structures*. Given journal publication requirements, as expected, nearly all of the JP RRs followed the traditional IMRaD report structure, a stronghold in academic publishing for the last 70 years [23]. IMRaD represents a pattern for structure more than the actual words covered by the abbreviation, and journals do not all follow a standard or uniform style. Nonetheless, IMRaD provides a level of uniformity in the way scientific evidence is presented [36]. In contrast, few NJP RRs reflected IMRaD, and instead, used graded entry formats, a combined graded entry frontend with an IMRaD backend or were part of multicomponent reports. What is unclear is the degree to which end-users informed these alternative formats identified, or if determined by an organizational mandate or what the producers thought was best. Collectively, this suggests a variety of formats are being used in the unpublished realm of RRs and underscores that groups are looking to alternative ways to organize content contained within a report. Although the use of IMRaD is engrained in journal publishing, it may be time to rethink whether this format is versatile or adaptable enough for new emerging research synthesis methods (e.g., rapid reviews).

*Considerations for decision-makers*. We found RRs published in journals were considerably shorter in page length and word count, a finding likely indicative of journal publishing restrictions. However, the main reports of the NJP RRs were more than double in length. Even though several NJP RRs used an alternative graded entry format, a lengthy report regardless of the structure may limit usability, and runs counter to evidence suggesting brief summaries are favoured among decision-makers [7, 12, 14]. Further, among both groups of RRs, the inclusion of key messages was relatively low. Recent findings indicate that decision-makers like having key messages upfront as part of a brief SR summary [37] and should be considered for all RRs, whether published or unpublished. Also, sections on equity, local applicability of results, and implementation considerations were not commonly included and only identified in NJP RRs. It may be JP RRs did capture such content, but that word restrictions limited the ability to publish full details. Nonetheless, given that many RRs are undertaken specifically for decision-making purposes, producers of RRs may be requested to include more details on actionable information (e.g., cost, training and resources required) to better support the application and implementation of findings. If so, such considerations should be thought through early in the process to best tailor RRs accordingly to meet the specific needs of decision-makers [1, 11, 37].

In terms of choice of font, JP RRs tended to use a serif font (e.g., Times Roman) for the main text while NJP RRs commonly used a sans serif font (e.g., Arial or Calibri). In print design, serif fonts are generally considered more readable than sans-serif fonts as the serifs reportedly serve aids readers moving from one letter to the next in a smoother fashion. However, differences in the legibility or reading speed of printed text between these fonts are negligible [38]. If reading electronic text, using sans serif typeface may improve reading time and accuracy [39]. Early research suggests that for alternative SR formats, use of certain sans serif fonts is preferred, and that reading materials on a computer is somewhat more favourable than print [29]. Whether these findings hold for RRs remains to be studied. However, knowing that certain fonts may be better suited for different mediums (e.g., print versus on screen) may be helpful in the design of future RR reports.

Specific to unpublished RRs, authorship or a corresponding author was not reported as part of the review identifying information in one-quarter of NJP RRs. Although all NJP RRs included a branded institutional logo, without an identifiable author, this could diminish the credibility of these reports. Also, over three-quarters of NJP RRs had no abstract (vs. 10% of JP RRs), and very few included an executive summary. A brief upfront summary would be beneficial given policymakers favour their use [11, 29, 37]. As well, the placement of a methods section for NJP RRs varied across reports in contrast to most JP RRs, where the methods sections followed the introduction as per IMRaD. Evidence suggests that methods details may not be as meaningful to decision-makers when compared to other included content [11, 29, 37, 40]. Nonetheless, from a reporting perspective, although a methods section does not necessarily need to be front and centre of a RR, these details need to be accessible somewhere in the report. Based on our entire sample, we encourage the improved use of a PRISMA flow diagram as part of the transparent reporting of methods.

### Directions for future research

We suggest exploring what content preferences exist for RRs. Beyond substance, we also need to evaluate our understanding of which design features are well received, in what contexts, and by whom. We need to develop RR prototypes and formally test usability to identify barriers and facilitators to their effective use. In particular, what remains unknown and requires further examination is the extent to which using IMRaD or alternative styles by end-users impacts perceived usefulness and levels of comprehension. Importantly, end-users (e.g., policymakers, clinicians, and patients) should drive this process of determining the most suitable formats as part of good knowledge translation practice. To fully assess the impact on uptake and use of RRs in decision-making, we must rigorously evaluate end-user format preferences, while also factoring in levels of health literacy and expertise in interpreting and using evidence among end-users. Given there is a general trend from print to electronic modes for receiving information, different mediums for delivering RR evidence should also be explored and take into account legibility, readability and aesthetic preferences. This study also highlights the need for producers of RRs to be transparent when reporting their review methods to facilitate quality assessment [41].

### Strengths and limitations

We used a broad working definition of RR and included RRs that addressed a variety of research questions beyond 'what works.' Thus, we erred on the side of inclusion, which may have resulted in a more heterogeneous set of RRs. However, we speculate that our findings are more broadly transferable and reflect the current state of RR methods in healthcare. To keep higher RR producing organizations from driving the results in the unpublished domain, we used a sampling approach aimed to control for potential clustering effect. In doing this, we increased the representativeness of our sample and overall generalizability. However, in taking this approach, we were unable to examine the full spectrum of RRs, primarily those from lower producing organizations. Therefore, our findings may not reflect the entire array of RR format and content features.

Further, although we noted whether RRs possessed certain features, we did not assess the quality of the characteristics, or whether the RRs were well conducted. Moreover, we only did a cursory examination of readability scores using one formulaic test. In the future, other readability measures, including reading time, amount recalled, and overall comprehension, would contribute to a more comprehensive evaluation of the text of RRs. Last, because we imposed language restrictions on our sample given resource limitations, our data set may be incomplete and likely does not reflect the entirety of RRs produced in 2016 in languages beyond English and French.

## Conclusions

Our findings highlight differences in certain format features between published and unpublished RRs, likely due to the use of distinct format structures (i.e., IMRaD use for journal articles while unpublished RRs tended to use alternative formats). There were also notable differences in labelled content likely driven in large part to the variances in format structures used. Our findings suggest that both sets of RRs may benefit from better use of plain language and more clear and concise reporting with a focus on key messages. Further, the information gleaned from the identified reports will directly inform those who conduct RRs. Importantly, this study provides a foundation for future research directed at better packaging of research results from RRs for policymakers and other key end-users to facilitate the uptake of evidence in policy and practice.

## Supporting information

S1 Checklist (PDF)

S1 File Common streamlined methods for rapid reviews. (PDF)

S2 File Types of graded entry formats. (PDF)

S3 File Full methods details. (PDF)

S4 File Search strategies. (PDF)

S5 File Eligibility criteria. (PDF)

S6 File Data collection forms. (PDF)

S7 File List of organizations producing rapid reviews included in the final non-journal published (NJP) sample. (PDF)

S1 Table Results presented in tables. (PDF)

S2 Table Peer review status and salient characteristics of potential predatory journals. (PDF)

## Abbreviations

RR: rapid review
SR: systematic review
IMRaD: introduction, methods, results, and discussion
GE: graded entry
MD: mean difference
OR: odds ratio
SD: standard deviation
MD: mean difference
OR: odd ratio
IQR: interquartile range
