# Supplementary material for: Assessing the format and content of journal published and non-journal published rapid review reports: A comparative study
Source: PLoS One. 2020 Aug 26;15(8):e0238025. doi: 10.1371/journal.pone.0238025 (PMC7449464; doi:10.1371/journal.pone.0238025)
Supplement: S1 Checklist — (PDF) [file pone.0238025.s001.pdf]

STROBE Statement—Checklist of items that should be included in reports of ***cross-sectional studies***

NOTE: There is no guideline specific to this type of comparative, methodological study. Therefore, we have used guidelines for reporting observational research as a proxy to ensure all relevant reporting has been included in the final manuscript. Specifically, we have followed the STROBE statement (for cross sectional studies) as a guide. Given this, we note that certain aspects of our research may not align and therefore, comply fully with the pre-specified checklist. Nonetheless, key elements of conduct have been reported on fully and transparently within the confines of the manuscript and appendices.

	**Item No**	**Recommendation**	**Reported on Page Number (#)**
**Title and abstract**	1	(*a*) Indicate the study's design with a commonly used term in the title or the abstract	Pg. 1 - comparative study is the term reported in the title
		(*b*) Provide in the abstract an informative and balanced summary of what was done and what was found	Pg. 3
**Introduction**			
Background/rationale	2	Explain the scientific background and rationale for the investigation being reported	Pg. 5
Objectives	3	State specific objectives, including any prespecified hypotheses	Pg. 6 – paragraph 1
**Methods**			
Study design	4	Present key elements of study design early in the paper	Pg. 7; S1-Appendix 1 – Full Methods Details
Setting	5	Describe the setting, locations, and relevant dates, including periods of recruitment, exposure, follow-up, and data collection	Pgs. 7-9; S1-Appendix 1 – Full Methods Details
Participants	6	(*a*) Give the eligibility criteria, and the sources and methods of selection of participants	Unit of analysis (rapid review reports) described – Pgs. 7-10; S1-Appendix 1 – Full Methods Details
Variables	7	Clearly define all outcomes, exposures, predictors, potential confounders, and effect modifiers. Give diagnostic criteria, if applicable	Pg. 9; S1-Appendix 1 – Full Methods Details (re: outcome variables)
Data sources/ measurement	8*	For each variable of interest, give sources of data and details of methods of assessment (measurement). Describe comparability of assessment methods if there is more than one group	*n/a*
Bias	9	Describe any efforts to address potential sources of bias	Pgs. 7-10; S1-Appendix 1- Full Methods Details; & Strength & Limitations section (pg. 24)
Study size	10	Explain how the study size was arrived at	Pgs. 7-10; S1-Appendix 1 – Full Methods Details
Quantitative variables	11	Explain how quantitative variables were handled in the analyses. If applicable, describe which groupings were chosen and why	Pgs. 8-10 – Data analysis section; S1-Appendix 1 – Full Methods Details

Statistical methods	12	(*a*) Describe all statistical methods, including those used to control for confounding	Pgs. 8-10; S1-Appendix 1 – Full methods Details
		(*b*) Describe any methods used to examine subgroups and interactions	Pgs. 10; S1-Appendix 1 Full Methods Details
		(*c*) Explain how missing data were addressed	n/a
		(*d*) If applicable, describe analytical methods taking account of sampling strategy	n/a
		(*e*) Describe any sensitivity analyses	n/a
**Results**			
Participants	13*	(a) Report numbers of individuals at each stage of study—eg numbers potentially eligible, examined for eligibility, confirmed eligible, included in the study, completing follow-up, and analysed	Pg. 11 – Results (paragraph 1) & Figure 1
		(b) Give reasons for non-participation at each stage	See Figure 1; Results pg. 11 paragraph 1
		(c) Consider use of a flow diagram	See Figure 1
Descriptive data	14*	(a) Give characteristics of study participants (eg demographic, clinical, social) and information on exposures and potential confounders	Pg. 11-12; Tables 1 & 2
		(b) Indicate number of participants with missing data for each variable of interest	Pg. 11; Tables 1-3
Outcome data	15*	Report numbers of outcome events or summary measures	Table 3; Figures 2-4
Main results	16	(*a*) Give unadjusted estimates and, if applicable, confounder-adjusted estimates and their precision (eg, 95% confidence interval). Make clear which confounders were adjusted for and why they were included	Pgs. 11-17; Table 3 (as reported in the methods section; estimated associations were crude and based on a univariate analysis and therefore, were not adjusted for other factors)
		(*b*) Report category boundaries when continuous variables were categorized	n/a
		(*c*) If relevant, consider translating estimates of relative risk into absolute risk for a meaningful time period	n/a
Other analyses	17	Report other analyses done—eg analyses of subgroups and interactions, and sensitivity analyses	n/a – as reported in Methods Section – planned subgroup analysis was not possible
**Discussion**			
Key results	18	Summarise key results with reference to study objectives	Pgs. 7-23; Conclusions Pg. 25
Limitations	19	Discuss limitations of the study, taking into account sources of potential bias or imprecision. Discuss both direction and magnitude of any potential bias	Pgs. 24-25
Interpretation	20	Give a cautious overall interpretation of results considering objectives, limitations, multiplicity of analyses, results from similar studies, and other relevant evidence	Pgs. 17-23

Generalisability	21	Discuss the generalisability (external validity) of the study results	Pg. 24-25 – Strengths & Limitations section
**Other information**			
Funding	22	Give the source of funding and the role of the funders for the present study and, if applicable, for the original study on which the present article is based	Pg. 26

*Give information separately for exposed and unexposed groups.

Note: An Explanation and Elaboration article discusses each checklist item and gives methodological background and published examples of transparent reporting. The STROBE checklist is best used in conjunction with this article (freely available on the Web sites of PLoS Medicine at http://www.plosmedicine.org/, Annals of Internal Medicine at http://www.annals.org/, and Epidemiology at http://www.epidem.com/). Information on the STROBE Initiative is available at www.strobe-statement.org.
