# Supplementary material for: Assessing the format and content of journal published and non-journal published rapid review reports: A comparative study
Source: PLoS One. 2020 Aug 26;15(8):e0238025. doi: 10.1371/journal.pone.0238025 (PMC7449464; doi:10.1371/journal.pone.0238025)
Supplement: S1 File — (PDF) [file pone.0238025.s002.pdf]

S1 File. Common Streamlined Methods for Rapid Reviews

Common Streamlined Methods for Rapid Reviews [18]

Protocol development

- Brief protocol is developed but usually not formally registered
- Generally, a restricted scope limited to one primary key question and critical outcomes only

Literature search

- Typically involves a search of a minimum of two key databases, with common limits on date, study design, setting, and language
- Limited grey literature searching (e.g., targeted search of key sources or websites)
- Use of study design search filters (i.e., for SRs or RCTs or) to facilitate staged screening
- Contacting authors for further information is uncommon
- Full text citations often limited to those electronically available

Screening

- Using a single reviewer, with or without verification by a second reviewer
- Emphasis placed on a staged screening process that focuses on higher level evidence (e.g., SRs) and if needed other designs (RCTs, non-RCTs, etc.)
- A targeted and iterative approach to screening that factors in study design and quality

Data extraction

- Using a single reviewer, with or without verification by a second reviewer
- Limit extraction to key study characteristics and outcomes

Risk of Bias Assessment

- Using a single reviewer, with or without verification by a second reviewer

Analysis

- May involve a cursory description of the studies or a more formal narrative synthesis; a formal quantitative synthesis (e.g., meta-analysis) will depend on time, resources, and appropriateness

Report production

- Use of an abbreviated and simplified report format; brief and useable for decision-makers
- Key messages and policy implications placed up front for the reader
- Peer review is not common
