# Supplementary material for: Assessing the format and content of journal published and non-journal published rapid review reports: A comparative study
Source: PLoS One. 2020 Aug 26;15(8):e0238025. doi: 10.1371/journal.pone.0238025 (PMC7449464; doi:10.1371/journal.pone.0238025)
Supplement: S2 File — (PDF) [file pone.0238025.s003.pdf]

S2 File. Types of Graded Entry Formats

Types of graded entry formats

1:3:25 format – a structure comprised of 1-page of main messages followed by a 3-page executive summary, with an additional 25 pages allotted for the main report including context, methods, main findings, and implications among information reported in clear, easy to understand language [27].

Inverted pyramid format – a structure that emphasizes the conclusions or key messages upfront followed by brief (executive) summary, followed by a lengthier report that provides specific details for the reader. For the purposes of this study, this format similarly follows a 1:3:25 format but does not strictly adhere to this page count.

SUPPORT Summary format – a structure developed to present the results of systematic reviews to decision-makers by first presenting key messages derived from findings, and generally include other components such as background information to provide context to the findings; a summary of the search approach and what was identified; a detailed summary of the main findings, including methodological quality of the evidence, and relevance to low and middle income countries including reference to applicability, impact on equity, economic considerations, need for monitoring and evaluation, and references [11].
