# Supplementary material for: Assessing the format and content of journal published and non-journal published rapid review reports: A comparative study
Source: PLoS One. 2020 Aug 26;15(8):e0238025. doi: 10.1371/journal.pone.0238025 (PMC7449464; doi:10.1371/journal.pone.0238025)
Supplement: S3 File — (PDF) [file pone.0238025.s004.pdf]

S3 File. Full Methods Details

Study Design

We conducted a descriptive, comparative study of a broad selection of RRs as the unit of analysis. This exploratory study was part of a suite of methodological work that stemmed from the same search strategy, sampling approach, and study selection process, but differed in purpose, data collection and analysis. Protocols for this study (https://osf.io/29xvk/) and two related investigations are available at: https://osf.io/v4k6f/; and https://osf.io/2av37/.

Defining 'format' and 'content'

For the purposes of this study, we defined *format or layout* to mean 'how' information was presented (i.e., the visual arrangement, appearance, or presentation of information contained within a report) with *content* referred to as the main features of a RR report in terms of 'what' information was presented (e.g., included sections or information).

Search strategy and process

Bibliographic searching to identify journal published (JP) RRs. A draft bibliographic database search strategy for MEDLINE was developed vis-à-vis key 'seed' articles by the contact investigators (CG and AS) and was peer-reviewed by a senior information specialist (BS) using the PRESS checklist [30]; the completed PRESS checklist can be found at: https://osf.io/29xvk/. The final MEDLINE search was modified for other bibliographic databases including the Ovid MEDLINE, Ovid MEDLINE In-Process and Other Non-Indexed Citations, Ovid EMBASE, Ebsco CINAHL, Proquest Educational Resources Information Center (ERIC), PsycINFO, and Wiley's The Cochrane Library (S). We did not apply language restrictions to the search strategy (See S4 File Search strategies).

Grey literature searching to identify non-journal published (NJP) RRs. Because a number of RRs are in the unpublished domain, we searched websites listed in CADTH's Grey Matters checklist [31] and the PROSPERO register (www.crd.york.ac.uk/PROSPERO/). Further, we searched the websites and a contact list of pre-identified organizations (n=148) that produce or commission RRs. If a rapid review did not report methodology or the reported methodology was unclear, we contacted authors for further information. As needed, we consulted as a proxy, any available internal methods guidance documents as requested and provided by authors/organizations.

Non-journal published (NJP) RRs sampling strategy. We identified a mix of higher and lower rapid review volume-producing organizations through grey literature searching efforts counting the number of rapid reviews that appeared to have been produced in 2016 and were available online. In this case, since a large proportion of identified RRs were likely to be clustered by organization, we expected that the format characteristics by organization would also be similar in most instances. Given this, we sampled proportionate to cluster size to create a sample in the NJP group that was generalizable to the RR literature. Knowing that at least some organizations produce more than one type of product that may be considered a RR (i.e., various series), we first catalogued the retrieved sample of NJP RRs by organization and then by product per organization.

For example, the Canadian Agency for Drugs and Technologies in Health (CADTH) has a rapid response service that produces more than one product type that would meet our eligibility criteria as a 'rapid review'. Next, we identified the total number of clusters from across all of the organizations and listed by size. Starting with the largest cluster, we then sampled from each proportionate to cluster size. In some cases, this meant that sampling took place at the organizational level and others within each RR type.

There was a substantially higher number of one rapid reviews product type from CADTH (i.e., CADTH summary with critical appraisal); a cluster that would have represented approximately 30% of the total number of included rapid reviews. To avoid overrepresentation of this CADTH rapid review product, we equalized the sample of this product with the next largest producing cluster, which for 2016 were rapid reviews produced as part of the pan-Canadian Oncology Drug Review (pCODR). With these adjusted totals, the grand total sum of rapid reviews across organizations was determined. The proportionate contribution of each cluster to the total was then calculated. Those proportionate contributions were then transposed using the journal-published group sample size as a guide. For the sake of feasibility, we strove for the same sample size between both groups, and only included those reports identified as being published in 2016.

Sample size

Given that this is a descriptive study and exploratory, no formal sample size calculation was required. However, we limited our sample for the sake of practicality using the aforementioned sampling strategy to ensure similar sized comparison groups.

Eligibility

Lacking a standard definition, we defined RRs as reports where the intent is to summarize evidence for use in any form of decision-making or information/decision support, directly or indirectly related to patient or healthcare, using systematic review methodology that is tailored to accommodate an expedited turnaround time [5, 15-16]. If authors did not cite or provide a definition of rapid review in their report for us to ascertain eligibility according to our definition, at a minimum they needed to provide a description to understand what they meant by a 'rapid' or accelerated feature of conduct (e.g., to meet a certain timeline, some type of modification of standard systematic review methodology). Systematic reviews that provided a description of 'rapid' conduct from a timing aspect and/or provided an explicit declaration to accelerate or abbreviate the systematic review process (even if not self-declared as rapid) were included. Reports were also included if authors simply stated 'rapid review' without further elaboration. Further, we did include reports without a specific methods section, as long as they otherwise met the definition. No maximum timeline of conduct was used for inclusion. Only RRs reported in English and French were considered. All types of RR research questions related to health care were also eligible. We did not include reports that only provided an annotated bibliography of relevant papers. The date of publication was used for determining the eligibility of published RRs. The date appearing in the report of unpublished RR (reasonably interpreted as the 'completion' date) was used as the best proxy for determining the finalization dates of those reports. For RRs that exist

both in the grey literature and in the published domain, the published version took precedent. A summary of the eligibility criteria is provided in S5.

Selection process

Citations and abstracts were downloaded and/or entered into a Reference Manager[1] database for de-duplication and uploaded to an internet-based systematic review program (DistillerSR[2]) to assess eligibility. Pilot testing of screening forms was done using a subset of 50 records for title/abstract screening, and 25 articles for full text screening. Titles and abstracts were reviewed by one person (CG/AS/KP/ZM); a second person (CG/AS) reviewed and verified all records excluded by the first reviewer. Full text reports of potentially relevant records were reviewed by two independent people (CG/AS/ZM/AB/NT), with disagreements resolved by consensus or a third person.

At the full text stage of screening, first we assessed the bibliographic results from the journal published domain to determine the sample size in the NJP group. Based upon this finding, we next determined how many RRs from the grey literature results were needed to create a similar sample size in the non-journal-published group. Any journal-published articles located during the search of grey literature sources were added to the database for inclusion in the journal-published group. We have documented reasons for exclusion of full text reports in a study flow diagram (Fig 1) that details the study selection process.

Data collection

A pre-specified data abstraction form was used to characterize the included RRs. We also extracted information specific to features of the reports across four broad categories considered to be involved in good document design, and that were most relevant given the nature of our study [32]. These included: 1) *report identifying information*; 2) *structure* (document organization); 3) *content* (bannered sections included); 4) *visual design* including i) legibility (i.e., font, spacing, background contracts); ii) graphic elements including typography (e.g., cues such as bolding, underlining, use of italics), graphic alternatives to text (e.g., use of tables, figures, lists, flowcharts, graphs), and iii) general layout including use of colour and branding. We also reported on *other factors* including the placement of certain sections in the report, how report format was decided, and whether or not stakeholders provided input on layout (See S6 data collection forms).

We pilot tested the forms using a subset of approximately 10 articles before implementation; forms were then revised accordingly before screening was started. For the general characteristics of the included RRs, one individual extracted data while a second person verified a minimum 10% random sample of studies. Format outcomes were extracted by one reviewer, with second reviewer providing full verification of all included RRs.

In addition, we did a cursory assessment of *readability* (or the ease with which the reader can understand written text) of the RRs using the Simple Measure of Gobbledygook (SMOG) readability test to estimates years of education a person needs to understand a piece of writing [33].

---

[1] Reuters T. Reference Manager 12 [Computer Program] New York: Thomson Reuters; 2011.
[2] Partners E. DistillerSR [Computer Program] Ottawa: Evidence Partners; 2011. www.evidencepartners.com

This tool has been used previously in studies assessing health information [34]. The SMOG test involves a readability formula that carries out calculations on a text, based primarily on sentence and word length, and results in a numerical score. Using an online SMOG calculator (https://www.learningandwork.org.uk/SMOG-calculator/smogcalc.php), we assessed the readability for the abstract, introduction (or background), and discussion (or conclusion) sections with scores corresponding to the level of education or reading age required to understand the analysed text. This involved one person (CB) cutting and pasting in sections of the specific sections into the software to generate scores, which were verified by a second reviewer (CG). We also did a *cursory word count* of reports (main body and the total length of document) by converting PDFs into Microsoft Word, and then using the word count feature (CG).

We also assessed whether or not peer review was conducted for each JP RR. To make this a more efficient process, we assumed that the majority of published articles would have undergone peer review. However, given the rise of illegitimate publishing entities, as a first step, we cross-checked each journal against the Directory of Open Access Journals (DOAJ) to confirm legitimacy of the publication (CG, MH). We further assessed each journal according to an evidence-based list of salient characteristics of predatory journals (CG, MH, AS, BA) [35]. This meant we reviewed the websites of journals and that of their corresponding publisher to review their respective policies including for peer review. For the NJP RRs, we noted if peer review was reported in the citation or if supplemental information obtained from the organization (e.g., methods guidance or website information) indicated peer review was part of their RR process (S8 Table).

Data analysis

The characteristics of each RR was reported in a series of tables and figures. For the main comparison (i.e., JP vs. NJP), we summarized characteristics using frequencies and/or proportions accompanied by appropriate statistical tests to determine if significant differences existed across variables between these groups in relation to their journal or non-publication status. To compare the features of both groups, we estimated the odds ratios based on conditional maximum likelihood method for binomial proportions (corresponding to the p-values from Fisher's exact test), and the mean differences for continuous variables (corresponding to the p-values from Welch's t-test) with 95% confidence intervals (CI). The estimated associations were crude and based on a univariate analysis and therefore, were not adjusted for other factors. For a subset of key features, we only reported on numerical differences between the JP and NJP RRs given any differences noted would likely be due to the distinct nature of biomedical journal publishing versus the in-house publishing structures of most healthcare research organizations producing RRs. Therefore, formal testing was only applied to a select group of variables where appropriate using a significance level of 0.05. Planned subgroup analyses (i.e., according to report structure, report production, purpose of the RR, timeframe of conduct, peer review status, and funding sources) were not possible due to insufficient data.

All analyses were overseen by the primary investigators with guidance from a Senior Biostatistician (WC). We used Microsoft Excel and R version 3.5.3 to calculate the statistics.[3]

Reporting guideline. Given no guideline specific to this methodological study type exists, to the extent possible we followed the STROBE Statement—Checklist for cross-sectional studies as a proxy.

[3] R Core Team. R: A language and environment for statistical computing. Vienna, Austria: R Foundation for Statistical Computing; 2013. http://www.R-project.org/.
