# Supplementary material for: Assessing the format and content of journal published and non-journal published rapid review reports: A comparative study
Source: PLoS One. 2020 Aug 26;15(8):e0238025. doi: 10.1371/journal.pone.0238025 (PMC7449464; doi:10.1371/journal.pone.0238025)
Supplement: S4 File — (PDF) [file pone.0238025.s005.pdf]

S4 File. Final Search Strategies

OVID

Database: Ovid MEDLINE(R) In-Process & Other Non-Indexed Citations and Ovid MEDLINE(R) <1946 to Present>, PsycINFO <1806 to February Week 2 2016>, Embase <1980 to 2016 Week 08>, ERIC <1965 to February 2016>
Search Strategy:
----
1 rapid review?.tw,kw.
2 expedited review?.tw,kw.
3 accelerated review?.tw,kw.
4 rapid literature review?.tw,kw.
5 rapid systematic review?.tw,kw.
6 rapid synthes*.tw,kw. (2169)
7 rapid evidence assessment?.tw,kw.
8 rapid evidence summary.tw,kw.
9 rapid evidence summaries.tw,kw.
10 technical brief?.tw,kw.
11 evidence brief?.tw,kw.
12 rapid technical brief?.tw,kw.
13 ultra rapid review?.tw,kw.
14 technote?.tw,kw.
15 rapid summary.tw,kw.
16 rapid summaries.tw,kw.
17 rapid critical appraisal?.tw,kw.
18 succinct evidence review?.tw,kw.
19 rapid approach*.tw,kw.
20 focused review?.tw,kw.
21 rapid product?.tw,kw.
22 summary review?.tw,kw.
23 targeted review?.tw,kw.
24 pragmatic review?.tw,kw.
25 rapid appraisal?.tw,kw.
26 rapid evidence appraisal?.tw,kw.
27 rapid knowledge synthes*.tw,kw.
28 rapid HTA?.tw,kw.
29 rapid health technology assessment?.tw,kw.
30 expedited synthes*.tw,kw.
31 expedited HTA?.tw,kw.
32 expedited health technology assessment?.tw,kw.
33 accelerated synthes*.tw,kw.
34 accelerated HTA?.tw,kw.
35 accelerated health technology assessment?.tw,kw.
36 mini synthes*.tw,kw.
37 mini HTA?.tw,kw.
38 mini health technology assessment?.tw,kw.
39 pragmatic synthes*.tw,kw.
40 pragmatic HTA?.tw,kw.
41 pragmatic health technology assessment?.tw,kw.
42 streamlined review?.tw,kw.
43 streamlined synthes*.tw,kw.
44 streamlined HTA?.tw,kw.
45 streamlined health technology assessment?.tw,kw.
46 snapshot review?.tw,kw.
47 snapshot synthes*.tw,kw.
48 snapshot HTA?.tw,kw.
49 snapshot health technology assessment?.tw,kw.
50 targeted synthes*.tw,kw.
51 targeted HTA?.tw,kw.
52 targeted health technology assessment?.tw,kw.
53 focused synthes*.tw,kw.
54 focused HTA?.tw,kw.
55 focused health technology assessment?.tw,kw.
56 preliminary review?.tw,kw.
57 preliminary synthes*.tw,kw.
58 preliminary HTA?.tw,kw.
59 preliminary health technology assessment?.tw,kw.
60 (rapid adj (meta-analy* or metanaly* or metaanaly* or met analy*)).tw,kw.
61 (expedited adj (meta-analy* or metanaly* or metaanaly* or met analy*)).tw,kw.
62 (accelerated adj (meta-analy* or metanaly* metaanaly* or met analy*)).tw,kw.
63 (mini adj (meta-analy* or metanaly* metaanaly* or met analy*)).tw,kw.
64 (pragmatic adj (meta-analy* or metanaly* metaanaly* or met analy*)).tw,kw.
65 (streamlined adj (meta-analy* or metanaly* metaanaly* or met analy*)).tw,kw.
66 (snapshot adj (meta-analy* or metanaly* metaanaly* or met analy*)).tw,kw.
67 (targeted adj (meta-analy* or metanaly* metaanaly* or met analy*)).tw,kw.
68 (focused adj (meta-analy* or metanaly* metaanaly* or met analy*)).tw,kw.
69 (preliminary adj (meta-analy* or metanaly* metaanaly* or met analy*)).tw,kw.
70 expedited systematic review?.tw,kw.
71 accelerated systematic review?.tw,kw.
72 mini systematic review?.tw,kw.

73 pragmatic systematic review?.tw,kw.
74 streamlined systematic review?.tw,kw.
75 snapshot systematic review?.tw,kw.
76 targeted systematic review?.tw,kw.
77 focused systematic review?.tw,kw.
78 preliminary systematic review?.tw,kw.
79 rapid systematic overview?.tw,kw.
80 expedited systematic overview?.tw,kw.
81 accelerated systematic overview?.tw,kw.
82 mini systematic overview?.tw,kw.
83 pragmatic systematic overview?.tw,kw.
84 streamlined systematic overview?.tw,kw.
85 snapshot systematic overview?.tw,kw.
86 targeted systematic overview?.tw,kw.
87 focused systematic overview?.tw,kw.
88 preliminary systematic overview?.tw,kw.
89 rapid evidence-based review?.tw,kw.
90 expedited evidence-based review?.tw,kw.
91 accelerated evidence-based review?.tw,kw.
92 mini evidence-based review?.tw,kw.
93 pragmatic evidence-based review?.tw,kw.
94 streamlined evidence-based review?.tw,kw.
95 snapshot evidence-based review?.tw,kw.
96 targeted evidence-based review?.tw,kw.
97 focused evidence-based review?.tw,kw.
98 preliminary evidence-based review?.tw,kw.
99 rapid evidence-based overview?.tw,kw.
100 expedited evidence-based overview?.tw,kw.
101 accelerated evidence-based overview?.tw,kw.
102 mini evidence-based overview?.tw,kw.
103 pragmatic evidence-based overview?.tw,kw.
104 streamlined evidence-based overview?.tw,kw.
105 snapshot evidence-based overview?.tw,kw.
106 targeted evidence-based overview?.tw,kw.
107 focused evidence-based overview?.tw,kw.
108 preliminary evidence-based overview?.tw,kw.
109 ((rapid or expedited or accelerated or mini or pragmatic or streamlined or snapshot or targeted or focused or preliminary) adj (evidence adj3 (review or reviews or overview*))).tw,kw.
110 ((rapid or expedited or accelerated or mini or pragmatic or streamlined or snapshot or targeted or focused or preliminary) adj "review of reviews").tw,kw.
111 or/1-110
112 limit 111 to yr="2013-current"
113 remove duplicates from 112
114 113 use emez
115 exp animal experimentation/ or exp models animal/ or exp animal experiment/ or nonhuman/ or exp vertebrate/
116 exp human/ or exp human experimentation/ or exp human experiment/
117 115 not 116
118 114 not 117
119 113 not 114
120 exp Animals/ not (exp Animals/ and Humans/)
121 119 not 120
122 news.pt.
123 121 not 122
124 118 or 123
125 remove duplicates from 124
126 125 use prmz
127 125 use emez
128 125 use eric
129 125 not (126 or 127 or 128)
****

Cochrane Library

Search Name: Rapid Reviews - Methods
Date Run: 22/02/16 19:51:06.418
Description: OHRI - 2016 Feb 19

ID Search Hits
#1 ("rapid review" or "rapid reviews"):ti,ab,kw
#2 ("expedited review" or "expedited reviews"):ti,ab,kw -
#3 ("accelerated review" or "accelerated reviews"):ti,ab,kw
#4 ("rapid literature review" or "rapid literature reviews"):ti,ab,kw
#5 ("rapid systematic review" or "rapid systematic reviews"):ti,ab,kw
#6 ("rapid synthesis" or "rapid syntheses"):ti,ab,kw
#7 ("rapid evidence" next assessment*):ti,ab,kw
#8 "rapid evidence summary":ti,ab,kw
#9 "rapid evidence summaries":ti,ab,kw
#10 ("technical brief" or "technical briefs"):ti,ab,kw
#11 ("evidence brief" or "evidence briefs"):ti,ab,kw
#12 ("rapid technical brief" or "rapid technical briefs"):ti,ab,kw
#13 ("ultra rapid review" or "ultra rapid reviews"):ti,ab,kw
#14 (technote or technotes):ti,ab,kw
#15 "rapid summary":ti,ab,kw
#16 "rapid summaries":ti,ab,kw
#17 ("rapid critical appraisal" or "rapid critical appraisals"):ti,ab,kw
#18 ("succinct evidence review" or "succinct evidence reviews"):ti,ab,kw
#19 ("rapid approach" or "rapid approaches"):ti,ab,kw
#20 ("focused review" or "focused reviews"):ti,ab,kw
#21 ("rapid product" or "rapid products"):ti,ab,kw
#22 ("summary review" or "summary reviews"):ti,ab,kw
#23 ("targeted review" or "targeted reviews"):ti,ab,kw
#24 ("pragmatic review" or "pragmatic reviews"):ti,ab,kw
#25 ("rapid appraisal" or "rapid appraisals"):ti,ab,kw
#26 ("rapid evidence appraisal" or "rapid evidence appraisals"):ti,ab,kw

#27 ("rapid knowledge synthesis" or "rapid knowledge syntheses"):ti,ab,kw
#28 ("rapid HTA" or "rapid HTAs"):ti,ab,kw
#29 ("rapid health technology assessment" or "rapid health technology assessments"):ti,ab,kw
#30 ("expedited synthesis" or "expedited syntheses"):ti,ab,kw
#31 ("expedited HTA" or "expedited HTAs"):ti,ab,kw
#32 ("expedited health technology assessment" or "expedited health technology assessments"):ti,ab,kw -
#33 ("accelerated synthesis" or "accelerated syntheses"):ti,ab,kw
#34 ("accelerated HTA" or "accelerated HTAs"):ti,ab,kw
#35 ("accelerated health technology assessment" or "accelerated health technology assessments"):ti,ab,kw
#36 ("mini synthesis" or "mini syntheses"):ti,ab,kw
#37 ("mini HTA" or "mini HTAs"):ti,ab,kw
#38 ("mini health technology assessment" or "mini health technology assessments"):ti,ab,kw
#39 ("pragmatic synthesis" or "pragmatic syntheses"):ti,ab,kw
#40 ("pragmatic HTA" or "pragmatic HTAs"):ti,ab,kw
#41 ("pragmatic health technology assessment" or "pragmatic health technology assessments"):ti,ab,kw
#42 ("streamlined review" or "streamlined reviews"):ti,ab,kw
#43 ("streamlined synthesis" or "streamlined syntheses"):ti,ab,kw
#44 ("streamlined HTA" or "streamlined HTAs"):ti,ab,kw
#45 ("streamlined health technology assessment" or "streamlined health technology assessments"):ti,ab,kw
#46 ("snapshot review" or "snapshot reviews"):ti,ab,kw
#47 ("snapshot synthesis" or "snapshot syntheses"):ti,ab,kw
#48 ("snapshot HTA" or "snapshot HTAs"):ti,ab,kw
#49 ("snapshot health technology assessment" or "snapshot health technology assessments"):ti,ab,kw
#50 ("targeted synthesis" or "targeted syntheses"):ti,ab,kw
#51 ("targeted HTA" or "targeted HTAs"):ti,ab,kw
#52 ("targeted health technology assessment" or "targeted health technology assessments"):ti,ab,kw
#53 ("focused synthesis" or "focused syntheses"):ti,ab,kw
#54 ("focused HTA" or "focused HTAs"):ti,ab,kw
#55 ("focused health technology assessment" or "focused health technology assessments"):ti,ab,kw
#56 ("preliminary review" or "preliminary reviews"):ti,ab,kw
#57 ("preliminary synthesis" or "preliminary syntheses"):ti,ab,kw
#58 ("preliminary HTA" or "preliminary HTAs"):ti,ab,kw
#59 ("preliminary health technology assessment" or "preliminary health technology assessments"):ti,ab,kw
#60 (rapid next (meta-analy* or metanaly* or metaanaly* or met analy*)):ti,ab,kw
#61 (expedited next (meta-analy* or metanaly* or metaanaly* or met analy*)):ti,ab,kw
#62 (accelerated next (meta-analy* or metanaly* or metaanaly* or met analy*)):ti,ab,kw
#63 (mini next (meta-analy* or metanaly* or metaanaly* or met analy*)):ti,ab,kw
#64 (pragmatic next (meta-analy* or metanaly* or metaanaly* or met analy*)):ti,ab,kw
#65 (streamlined next (meta-analy* or metanaly* or metaanaly* or met analy*)):ti,ab,kw
#66 (snapshot next (meta-analy* or metanaly* or metaanaly* or met analy*)):ti,ab,kw
#67 (targeted next (meta-analy* or metanaly* or metaanaly* or met analy*)):ti,ab,kw
#68 (focused next (meta-analy* or metanaly* or metaanaly* or met analy*)):ti,ab,kw
#69 (preliminary next (meta-analy* or metanaly* or metaanaly* or met analy*)):ti,ab,kw
#70 ("expedited systematic review" or "expedited systematic reviews"):ti,ab,kw
#71 ("accelerated systematic review" or "accelerated systematic reviews"):ti,ab,kw
#72 ("mini systematic review" or "mini systematic reviews"):ti,ab,kw
#73 ("pragmatic systematic review" or "pragmatic systematic reviews"):ti,ab,kw
#74 ("streamlined systematic review" or "streamlined systematic reviews"):ti,ab,kw
#75 ("snapshot systematic review" or "snapshot systematic reviews"):ti,ab,kw
#76 ("targeted systematic review" or "targeted systematic reviews"):ti,ab,kw
#77 ("focused systematic review" or "focused systematic reviews"):ti,ab,kw
#78 ("preliminary systematic review" or "preliminary systematic reviews"):ti,ab,kw
#79 ("rapid systematic overview" or "rapid systematic overviews"):ti,ab,kw
#80 ("expedited systematic overview" or "expedited systematic overviews"):ti,ab,kw
#81 ("accelerated systematic overview" or "accelerated systematic overviews"):ti,ab,kw
#82 ("mini systematic overview" or "mini systematic overviews"):ti,ab,kw
#83 ("pragmatic systematic overview" or "pragmatic systematic overviews"):ti,ab,kw
#84 ("streamlined systematic overview" or "streamlined systematic overviews"):ti,ab,kw
#85 ("snapshot systematic overview" or "snapshot systematic overviews"):ti,ab,kw

#86 ("targeted systematic overview" or "targeted systematic overviews"):ti,ab,kw
#87 ("focused systematic overview" or "focused systematic overviews"):ti,ab,kw
#88 ("preliminary systematic overview" or "preliminary systematic overviews"):ti,ab,kw
#89 ("rapid evidence-based review" or "rapid evidence-based reviews"):ti,ab,kw
#90 ("expedited evidence-based review" or "expedited evidence-based reviews"):ti,ab,kw
#91 ("accelerated evidence-based review" or "accelerated evidence-based reviews"):ti,ab,kw
#92 ("mini evidence-based review" or "mini evidence-based reviews"):ti,ab,kw
#93 ("pragmatic evidence-based review" or "pragmatic evidence-based reviews"):ti,ab,kw
#94 ("streamlined evidence-based review" or "streamlined evidence-based reviews"):ti,ab,kw
#95 ("snapshot evidence-based review" or "snapshot evidence-based reviews"):ti,ab,kw
#96 ("targeted evidence-based review" or "targeted evidence-based reviews"):ti,ab,kw
#97 ("focused evidence-based review" or "focused evidence-based reviews"):ti,ab,kw
#98 ("preliminary evidence-based review" or "preliminary evidence-based reviews"):ti,ab,kw
#99 ("rapid evidence-based review" or "rapid evidence-based reviews"):ti,ab,kw
#100 ("expedited evidence-based review" or "expedited evidence-based reviews"):ti,ab,kw
#101 ("accelerated evidence-based review" or "accelerated evidence-based reviews"):ti,ab,kw
#102 ("mini evidence-based overview" or "mini evidence-based overviews"):ti,ab,kw
#103 ("pragmatic evidence-based overview" or "pragmatic evidence-based overviews"):ti,ab,kw
#104 ("streamlined evidence-based overview" or "streamlined evidence-based overviews"):ti,ab,kw
#105 ("snapshot evidence-based overview" or "snapshot evidence-based overviews"):ti,ab,kw
#106 ("targeted evidence-based overview" or "targeted evidence-based overviews"):ti,ab,kw
#107 ("focused evidence-based overview" or "focused evidence-based overviews"):ti,ab,kw
#108 ("preliminary evidence-based overview" or "preliminary evidence-based overviews"):ti,ab,kw
#109 ((rapid or expedited or accelerated or mini or pragmatic or streamlined or snapshot or targeted or focused or preliminary) next (evidence near/3 (review or reviews or overview*))):ti,ab,kw
#110 ((rapid or expedited or accelerated or mini or pragmatic or streamlined or snapshot or targeted or focused or preliminary) next "review of reviews"):ti,ab,kw
#111 {or #1-#110} Publication Year from 2013 to 2016

DSR
DARE
CENTRAL
HTA

Note: CINAL search available upon request
