# Supplementary material for: Assessing the format and content of journal published and non-journal published rapid review reports: A comparative study
Source: PLoS One. 2020 Aug 26;15(8):e0238025. doi: 10.1371/journal.pone.0238025 (PMC7449464; doi:10.1371/journal.pone.0238025)
Supplement: S5 File — (PDF) [file pone.0238025.s006.pdf]

S5 File. Eligibility Criteria

- We defined RRs as reports where the intent is to summarize evidence for use in any form of decision-making or information/decision support, directly or indirectly related to patient or healthcare, using SR methodology that is tailored to accommodate an expedited turnaround time [5, 15, 16].
- If authors did not cite or provide a definition of RR, at a minimum they needed to provide a description that they understood what they meant by a 'rapid' or accelerated feature of conduct (e.g., to meet a certain timeline, some type of modification of standard systematic review methodology).
- SRs that provided a description of 'rapid' conduct from a timing aspect and/or provided an explicit declaration to accelerate or abbreviate the SR process (even if not self-declared as rapid) were included.
- We included reports if authors simply stated 'rapid review' without further elaboration.
- We did include reports without a specific methods section, as long as they otherwise met the definition.
- No maximum timeline of conduct was used for inclusion.
- Only RRs reported in English and French were included.
- All types of RR research questions related to health care were eligible.
- We did not include reports that only provided an annotated bibliography of relevant papers.
