# Supplementary material for: Assessing the format and content of journal published and non-journal published rapid review reports: A comparative study
Source: PLoS One. 2020 Aug 26;15(8):e0238025. doi: 10.1371/journal.pone.0238025 (PMC7449464; doi:10.1371/journal.pone.0238025)
Supplement: S6 File — (PDF) [file pone.0238025.s007.pdf]

S6 File. Data Collection Forms

General Study Characteristics

1. Please enter the REFID [enter]

2. Year of publication or report date [enter]

3. Is a list of authors provided?
   - o Yes (specify #)
   - o No

4. Corresponding author listed
   - o Yes
   - o No

5. Was this RR produced by a specific organization, institute, centre, research team, department etc.? (please specify)
   - o Yes (please specify producer)
   - o No

6. Country of corresponding author or producer [enter]

7. Was this RR specifically commissioned/requested?
   - o Yes (please specify commissioner/requester)
   - o No - does not appear to be commissioned/requested by a specific source

8. Country of the commissioner/requestor [please enter]

9. Enter the language of rapid review report:
   - o English
   - o French
   - o Other (please specify)

10. Please describe the RR report as one of the following:
    - o Journal published report
    - o Non-journal published (publicly available)
    - o Non-journal published (not publicly available)

11. Did the authors report on 'funding' in the RR?
    - o Yes [please specify]
    - o No, not reported

12. Was this report peer-reviewed?
    - o Yes - grey lit report
    - o No, not peer-reviewed - grey lit report
    - o Not reported - grey lit report

- Journal published report
- Unclear

13. Was the purpose, reason, rationale for doing the RR clearly stated by the authors? (Please select an answer)

- Yes, clearly stated by the authors
- No, not specifically stated

14. How much time did it take to conduct the RR report: [enter dates, numbers of weeks/months or if not reported]

15. How many key questions did the RR address? [enter]

16. How many reports/studies were included in this RR? [enter]

17. Number of study designs included in the RRs [enter]

18. Types of study designs included (check all that apply)

- ☐ RCTs
- ☐ Observational studies
- ☐ Systematic reviews
- ☐ Other
- ☐ Unclear

19. Please indicate which category best fits the main intervention/exposure of the RR?

- Pharmacological
- Non-pharmacological
- Mixed
- Not addressing intervention/exposure

20. Broad ICD-10 category of the topic investigated in this rapid review? (Please check off the 'best suited' broad category for each RR report; may select up to a few categories)

For guidance, see http://apps.who.int/classifications/icd10/browse/2016/en. Please choose the most relevant disease(s) according to the ICD-10 system (2016) Please choose the MOST relevant categories. (e.g. if the condition is lung cancer, please select "Neoplasms" and "Diseases of the respiratory system").
If none of the clinical topics are relevant - please select (Not applicable) - and briefly specify why

- ☐ Infections and parasitic diseases
- ☐ Neoplasms (incl. cancers, carcinomas, tumors)
- ☐ Diseases of the blood and blood forming organs, immune mechanism
- ☐ Endocrine, nutritional, and metabolic disease
- ☐ Mental and behaviour disorders
- ☐ Diseases of the nervous system
- ☐ Diseases of the eye and adnexa
- ☐ Diseases of the ear and mastoid process
- ☐ Diseases of the circulatory system
- ☐ Diseases of the respiratory system
- ☐ Diseases of the digestive system

- ☐ Diseases of the skin and subcutaneous tissue
- ☐ Diseases of the musculoskeletal system and connective tissue
- ☐ Diseases of the genitourinary system
- ☐ Pregnancy, childbirth, and the puerperium
- ☐ Certain conditions originating in the perinatal period
- ☐ Congenital malformations, deformations, and chromosomal abnormalities
- ☐ Symptoms, signs and abnormal clinical and laboratory findings, not elsewhere classified
- ☐ Injury, poisoning, and certain other consequences of external causes
- ☐ External causes of morbidity and mortality
- ☐ Factors influencing health status and contact with health services
- ☐ Unclear (please specify)
- ☐ Not applicable (please explain)
- ☐ Other (please specify)

21. End-user consultations during development of the RR reported?

    - o Yes
    - o No
    - o No, not reported

Formatting Characteristics

1. Does the document contain a cover or title page?

    - o Yes
    - o No

2. Is a publication date (i.e., the Year Published) reported in the document?

    - o Yes, within the front end of the document only
    - o Yes, cited throughout the document (as is often the case with journal publications - cited on each or every 2nd pg)
    - o Partially visible/reported
    - o Yes, other (please specify)
    - o No, publication date is not cited

3. Was authorship cited in the document?

    - o Yes, placed in usual byline under the title (as for most journal articles)
    - o Yes, placed on the title page of the report
    - o Yes, placed near and/or at end of the main document
    - o Yes, listed in other location (please describe)
    - o No specific authorship was listed/provided

4. Page numbering throughout the document?

    - o Yes
    - o No

5. Does this document contain a section entitled, "Executive Summary"?

    - o Yes

- No

6. Length of the RR document?

   - Total number of pages [please enter a #]
   - For non-journal published - length of the abstract summary (if applicable) [enter a #]
   - For non-journal published - length of the executive summary (if applicable) [enter a #]
   - For all documents, length of the main report only [please enter a #]

7. Type of format the RR document most closely resembles:

   See sheet of definitions

   - Traditional scientific IMRAD format (intro, methods, results, analysis, discussion)
   - Graded entry: SUPPORT Style
   - Graded entry: 1:3:25 (adherence to pg counts)
   - Graded entry: Inverted pyramid (similar to 1:3:25 but doesn't strictly adhere to pg counts)
   - Graded entry (front end) combined with IMRAD
   - Other [please describe]

8. Please indicate which of the following IMRAD sections are bannered/visible components within the main RR document/report? (Check all that apply)

   If synonyms are used for any of the headers listed here, please check off 'other' and indicate the header in the textbox (e.g. Summary and Implications)

   - ☐ Abstract or Summary
   - ☐ Introduction or Background or both section
   - ☐ Methods section
   - ☐ Results or Findings section
   - ☐ Discussion section
   - ☐ Conclusions section
   - ☐ None of the above
   - ☐ Other (please specify)

9. Are any of the following sections included/bannered in the main RR document? (Check all that apply)

   Looking for A MAJOR HEADING OR SUBHEADING
   - if outside of the list provided, please select "Other - Various"

   If synonyms are used for any of the listed headers, please check off the appropriate header and indicate the synonym used in the textbox. For example, authors may use "economic evaluation" instead of "cost implications". Please check off "cost implications" and specify "economic evaluation" in the textbox.

   - ☐ Key messages
   - ☐ Disclaimer
   - ☐ Policy options or implications
   - ☐ Cost implications/economic analysis
   - ☐ Limitations
   - ☐ Recommendations for future research
   - ☐ References
   - ☐ Appendices (or Supplemental Files) - links to
   - ☐ Acknowledgements

- ☐ Competing Interests/Conflicts of Interest
- ☐ Authors' Contributions
- ☐ Abbreviations
- ☐ Implications
- ☐ Other various headings throughout
- ☐ None of the above (not applicable)

10. Are any of the following formally 'bannered' in the main document? (Check all the apply)

    These are NOT SO COMMON sections that may appear in some reports. However, included as important for applicability/localization of information (important to healthcare manager/policymakers)
    In either Major Headings or Subheadings?

    - ☐ Quality of the body of evidence
    - ☐ Equity
    - ☐ Local applicability of the results
    - ☐ Implementation
    - ☐ None of the above (not applicable)
    - ☐ Other (misc. not reported above)

11. The RR document appears to have been published:
    - o Using a 'high quality' typesetting system (La Tex; Python) - used in standard technical and academic publications; likely to apply to journal articles and professionally developed reports
    - o Using a word processing system/desktop publishing software (i.e., looks like a formatted word document/perhaps converted to PDF); likely only applicable to certain RR reports but some journal articles may appear in prepublication form as word docs

12. What was the general typeface of the titles/headers?
    - o Serif (times roman, cambria, georgia etc.)
    - o San Serif (arial, calibri etc.)

13. What was the general typeface of the of the main text of the document? (See above for reference)
    - o Serif (times roman, cambria, georgia etc.)
    - o Sans Serif (arial, calibri etc.)

14. What was the general typeface of the abstract text?
    - o Serif (times roman, cambria, georgia etc.)
    - o Sans Serif (arial, calibri etc.)

15. What was the general typeface of the abstract headers?
    - o Serif (times roman, cambria, georgia etc.)
    - o Sans Serif (arial, calibri etc.)

16. Please describe the line spacing of the main document?
    - o Appeared to be single spaced
    - o Appeared to use line spacing beyond single spaced
    - o Not applicable for published journal articles that appear to have used a high-quality typesetting

17. How was the information in the main document presented?

- ○ single column
- ○ double column (side by side)
- ○ other (please specify)

18. Which of the following typographic cues are used to help readers focus in on certain pieces of information in the RR document? (Check all the apply)

*Please note this includes any section of the main report (excluding references and appendices) - BE MINDFUL OF italics, bold, underline, bullets etc.

- ☐ Use of bolder headers
- ☐ Use of bolded 'text' or key words/phrases
- ☐ Use of coloured 'text' or keywords/phrases
- ☐ Use of underlining 'text' or keywords/phrases or titles
- ☐ Bullet lists
- ☐ Call-out boxes
- ☐ None of the above
- ☐ Use of Italics to emphasize key words or phrases

19. Was the main document TEXT presented in monochrome or colour? Ie. Use of black and white text or use of colour text (not including hyperlinks or figures).

monochrome = black & white, or use of grey scale only; Journal cover page may contain colour and the journal logo (please do not count this); look to the subsequent pages to see if presented in any other colour scheme other than black and white (mono).

- ○ Colour
- ○ Mono (black and white)

20. Specify background colour: [enter]

21. Specify main text colour Colour: [enter]

22. Any accent colours used in the documents? (Please specify)

- ☐ Blue
- ☐ Green
- ☐ Teal
- ☐ Purple
- ☐ Other
- ☐ Mix

23. Were tables presented in the main document? (excluding appendices)

**Do not double count duplicate tables - for example, if the same table appears in the Executive Summary and in the main report, count this table only once.

- ○ Yes (please specify how many - #)
- ○ No

24. Types of tables presented:

- ☐ Table of study characteristics
- ☐ Table of study characteristics
- ☐ List of excluded studies
- ☐ Search strategies
- ☐ Sample screening forms
- ☐ Data extraction forms
- ☐ Outcome specific data tables
- ☐ Evidence tables (for each study)
- ☐ Quality assessment
- ☐ GRADE summary of findings
- ☐ Other (i.e., non study level data/information tables for ex. in the intro or background section or methods etc.) [please describe]

25. Materials were provided in the appendices?

- ☐ PRISMA Checklist
- ☐ Study Protocol
- ☐ Other 1 (please specify)
- ☐ Other 2 (please specify)
- ☐ Other 3 (please specify)
- ☐ No tables provided in appendices
- ☐ Not applicable
- ☐ Appendices not freely accessible; could not assess

26. Was a PRISMA flow diagram included in the body of the RR report?

- ○ Yes, included in the body of the report
- ○ No, but a flow diagram was included in the appendices or supplemental material linked to this report
- ○ No diagram was provided

27. Were figures included in the main document? (excluding appendices)

- ○ Yes (please describe if forest, funnel or other; how many - #)?
- ○ No

28. Please indicate which of the following types of figures were included in APPENDICES? Check all that apply

Figures = charts, graphs, diagrams

- ☐ PRISMA Flow diagram
- ☐ Forest plot
- ☐ Funnel plot
- ☐ Other (e.g., drawings etc.)
- ☐ No figures provided in appendices
- ☐ Not applicable
- ☐ Appendices not freely accessible; could not assess

29. Are there any institutional logos presented in the document?

- Yes
- No
- N/A (for journal publications)

30. Any additional visual cues or institutional taglines used in the document that brand the organization commissioning or producing the RR document?
    - Yes (please specify) [logo, header or footer text, other]
    - No

31. Placement of the methods section?
    - Front end of the document (e.g., for most journal publications
    - Back end
    - In the general text; no specific section header
    - Methods information in the appendices
    - Other

32. Placement of the 'key messages'?
    - Front end of the document
    - Back end of the document
    - Other

33. Placement of 'disclaimer'?
    - Front end of the document
    - Back end of the document
    - Other

34. Who determined format of the report?

    For non-journal published RRs, we will not make any assumptions. If not explicitly reported in the text, select "Unclear/Can't tell".
    - Journal publisher (not applicable for non-journal published citations)
    - Producer of the RR (research team drives the template vs. commissioner)
    - Commissioner of the RR report (requester drives the research template)
    - Unclear/Can't tell

35. In relation to formatting, was there engagement beyond the research team of stakeholders (e.g., commissioners; end-users etc. Stakeholders intended as a very broad term in this context) with regards to the look/layout of the final end-product?
    - Yes
    - No
    - Not reported
    - N/A for journal publications

36. Do authors make mention of additional materials that are available upon request? *(beyond what is available in appendices or links to supplemental files)
    - Yes, mentioned or listed (please specify what is available)
    - Not listed or mentioned in the report

37. Publication Information:

Select "None of the above are applicable" for non-journal publishes reports

- States "Open Access" (Journal published only)
- None of the above are applicable

38. SMOG Score Information [entered into a separate spreadsheet]

39. Word counts [entered into a separate spreadsheet]
