# Supplementary material for: Assessing the format and content of journal published and non-journal published rapid review reports: A comparative study
Source: PLoS One. 2020 Aug 26;15(8):e0238025. doi: 10.1371/journal.pone.0238025 (PMC7449464; doi:10.1371/journal.pone.0238025)
Supplement: S7 File — (PDF) [file pone.0238025.s008.pdf]

S9 File. List of organizations producing rapid reviews included in the final non-journal published (NJP) sample:

Listed in alphabetical order:

Adelaide Health Technology Assessment, University of Adelaide

Australian Safety and Efficacy Register of New Interventional Procedures – Surgical (ASERNIP-S)

CADTH Rapid Response Program

Deakin University's Centre for Social and Early Development (SEED)

Evidence for Policy and Practice Information and Co-ordinating Centre (EPPI-Centre)

Evidence-based Synthesis Program (ESP) Coordinating Center Portland VA Health Care System

Healthcare Improvement Scotland

HealthPACT

Institut national d'excellence en santé et services sociaux

Institute for Clinical and Economic Review (ICER)

Institute of Health Economics (IHE)

Knowledge Synthesis Group, Ottawa Hospital Research Institute

McMaster Health Forum

Newfoundland & Labrador Centre for Applied Health Research (NLCAHR)

Ontario Drug Policy Research Network (ODPRN)

OHTN Rapid Response Service (Ontario HIV Treatment Network)

pan-Canadian Oncology Drug Review (CADTH) (pCODR)

Penn Medicine's Center for Evidence-based Practice

Program in Evidence-Based Care (PEBC), Cancer Care Ontario (CCO)

Public Health Wales Observatory

RAND Southern California Evidence-based Practice Center

Region of Peel (Public Health)

Sax Institute

Vanderbilt Evidence-based Practice Center

WHO (acting as host organization for, and secretariat of the European Observatory on Health Systems and Policies)
