# Supplementary material for: Assessing the format and content of journal published and non-journal published rapid review reports: A comparative study
Source: PLoS One. 2020 Aug 26;15(8):e0238025. doi: 10.1371/journal.pone.0238025 (PMC7449464; doi:10.1371/journal.pone.0238025)
Supplement: S1 Table — (PDF) [file pone.0238025.s009.pdf]

S7 Table 1. General Characteristics of Included Rapid Reviews (RRs)

Characteristics	Journal (n=52)	Non-journal (n=51)
List of authors provided, n (%)	52 (100)	37 (73)
Number of authors listed, mean (SD)	5.5 (3.5)	3.9 (2.5)
Corresponding authors listed, n (%)	46 (88)	3 (6)
Country of corresponding author or producer, n (%)		
United Kingdom	20 (38)	1 (2)
Canada	12 (23)	30 (59)
Australia	4 (8)	10 (20)
United States	3 (6)	7 (14)
Italy	2 (4)	0
Belgium	2 (4)	0
Scotland	1 (2)	2 (4)
Denmark; Spain; China; Sweden; Saudi Arabia; Germany; Taiwan; Netherlands[1]	1 (2)	0
Other	0	1 (2)
RRs produced by a specific organization/institute/team, n (%)	7 (13)	51 (100)
Unique organizations, n	5	25
Academic affiliation, n (%)		
Yes[2]	50 (96)	17 (33)
No	1 (2)	31 (61)
Unclear	1 (2)	3 (6)
RR commissioned or requested, n (%)	13 (25)	27 (53)
Country, n		
United Kingdom	7	1
Canada	3	9
Denmark	1	0
Belgium	1	0
Australia	1	10
United States	0	4
Scotland	0	2
Not reported/Unclear	0	1
Reported funding, n (%)	39 (75)	28 (55)
Funding source, n		
External, peer reviewed grant	6	2
External, non-commercial (fee for service)	22	25
External, commercial (fee for service)	2	0
Internal	0	1
Specified no funding received	9	0
Type of publishing journal, n (%)		
General	15 (29)	n/a
Specialty	37 (71)	n/a
RRs Publicly available, n (%)	36 (69)	50 (98)
Unique journals, n	47	n/a
Peer reviewed, n (%)	50 (96)[3]	6 (12)[4]
Language of the RRs, n (%)		
English	52 (100)	50 (98)
French	0	1(2)

SD – standard deviation; n/a – not applicable

[1] Per country

[2] First, second, corresponding, or senior author

[3] Peer review confirmed if journal listed on the DOAJ or if specifically stated as a policy of the journal

[4] NJP peer review status based on reporting of methods or from available methods guidance from respective institutions

S7 Table 2. General Content of the Identified Rapid Reviews (RRs)

Content	Journal (n=52)	Non-journal (n=51)
Purpose or rationale for RR clearly stated by the authors	33 (63)	30 (59)
Number of RRs that did not report time to conduct, n (%)	49 (94)	48 (94)
Time to conduct the RR reported, n (%)	3 (6)	3 (6)
4 weeks	0	2
8 weeks	1	0
17 weeks	0	1
24 weeks	1	0
32 weeks	1	0
Key questions, n (%)		
1	37 (71)	31 (61)
2	7 (13)	8 (16)
3	3 (6)	8 (16)
≥4	5 (10)	4 (8)
Number of included studies in the RRs, median (IQR) [range]	55 (30) [2-1820]	9 (23) [1-147]
Number of study designs included in the RRs, n (%)		
One	14 (27)	23 (45)
Two	12 (23)	10 (20)
Three	19 (37)	14 (27)
Four	4 (8)	3 (6)
Five	3 (6)	1 (2)
RRs with only one study design, n		
RCTs	2	9
Observational studies (cohorts, case-control, cross-sectional)	4	6
Systematic Reviews	3	6
Other[1]	1	2
Unclear	4	0
Frequency of included study designs, n		
Systematic reviews	15	25
RCTs	17	24
Observations studies (cohorts, case-control, cross-sectional)	36	25
Other[1]	21	16
Unclear	28	12
Main intervention, n (%)		
Pharmacological	4 (8)	13 (25)
Non-pharmacological	29 (56)	28 (55)
Mixed	1 (2)	4 (8)
Other (does not address an intervention or exposure)	18 (35)	6 (12)
Broad ICD-10 category[2]		
1 category addressed	28 (54)	26 (51)
>2 categories addressed	24 (46)	25 (49)
End-user consultations during development of the RR, n (%)	18 (35)	29 (57)

IQR – inter-quartile range

[1] Other may include qualitative, quasi-experimental designs including interrupted time series, controlled before/after, case series etc.

[2] ICD-10 - International Classification of Diseases, Tenth Revision

S7 Table 3. 2016 Rapid Reviews: Report Format, Layout, Content and Other Features

	Journal (N=52)	Non-journal (N=51)	Odds Ratio (95% CI)	P-value, F/W[1]
**Report identifying information**				
Title or cover page, n (%)	2 (4)	35 (69)	Not tested	
Authorship reported, n (%)	**52 (100)**	**37 (73)**	OR not available	< 0.0001, F
In the byline under the title (as per most journals)	43 (83)	3 (6)		
On the title or cover page	2 (4)	5 (10)		
Listed at/near end of the main document	0	7 (14)		
Other	7 (13)	22 (42)		
Publication date visible/reported, n (%)	52 (100)	51 (100)	Not tested	
Cited at the front end of the document	15 (29)	27 (53)		
Cited throughout the document	32 (62)	24 (47)		
Partially visible/reported	5 (10)	0		
**Structure (document organization)**				
Type of report structure, n (%)				
Traditional IMRAD[2]	**48 (92)**	4 (8)	**125.49 (28.75-792.06)**	< 0.0001, F
Graded entry[3]	0	24 (47)		
1:3:25[4]	0	1		
SUPPORT Summary Style[5]	0	4		
Inverted Pyramid[6]	0	3		
Graded entry (other)	0	16		
Graded entry front end followed by IMRAD	2 (4)	11 (22)		
Multicomponent report[7]	2 (4)	12 (24)		
Page numbering in the document, n (%)	52 (100)	48 (94)	Not tested	
Page length, mean (SD)			Not tested	
Full report (all pages)	20.77 (33.56)	50.84 (51.18)		
Main report only[8]	12.17 (10.40)	27.14 (25.22)		
Executive Summary	4.5 (2.1) (n=2)	8.5 (10.25) (n=18)		
**Content**				
Banner and headers, n (%)[9]				
Abstract	**47 (90)**	12 (24)	**29.10 (9.01-115.99)**	< 0.0001, F
Executive Summary	2 (4)	**11 (22)**	**0.15 (0.02-0.74)**	0.01, F
Introduction/background	43 (83)	36 (71)	1.98 (0.72-5.78)	0.17, F
Methods	**51 (98)**	42 (82)	**10.73 (1.39- 487.01)**	0.01, F
Results	48 (92)	46 (90)	1.30 (0.26-6.98)	0.74, F
Discussion	**45 (87)**	6 (12)	**45.14 (13.39-184.65)**	< 0.0001, F
Conclusions	**45 (87)**	18 (35)	**11.45 (4.07-36.59)**	< 0.0001, F
Key Messages	3 (6)	**24 (47)**	**0.07 (0.01-0.26)**	< 0.0001, F
Disclaimer	3 (6)	**26 (51)**	**0.06 (0.01-0.22)**	< 0.0001, F

	Journal (N=52)	Non-journal (N=51)	Odds Ratio (95% CI)	P-value, F/W[1]
Policy options/implications	1 (2)	**20 (39)**	**0.03 (0.00-0.21)**	< 0.0001, F
Cost implications	3 (6)	**23 (45)**	**0.08 (0.01-0.27)**	< 0.0001, F
Limitations	24 (46)	18 (35)	1.56 (0.66-3.75)	0.32, F
Recommendations for future research	6 (12)	3 (6)	2.07 (0.41-13.56)	0.49, F
References	50 (96)	51 (100)	0.00 (0.00-5.41)	0.50, F
Appendices	20 (38)	**35 (69)**	**0.29 (0.12-0.69)**	0.01, F
Acknowledgement	**26 (50)**	13 (25)	**2.89 (1.18-7.36)**	0.01, F
Conflict of Interests	**37 (71)**	10 (20)	**9.84 (3.73-28.21)**	< 0.0001, F
Author contributions	**20 (38)**	2 (4)	**14.95 (3.28-140.57)**	< 0.0001, F
Abbreviations	8 (15)	10 (20)	0.75 (0.23-2.34)	0.61, F
Implications (not otherwise specified)	5 (10)	3 (6)	Not tested	
Quality of the body of evidence	1 (2)	3 (6)	Not tested	
Equity	0	2 (4)	Not tested	
Local applicability of results	0	5 (10)	Not tested	
Implementation considerations	0	3 (6)	Not tested	
Various (miscellaneous – other)	50 (96)	50 (98)	Not tested	
**Visual design**				
Legibility				
Document preparation system, n (%)				
High quality typesetting system	48 (92)	12 (24)	Not tested	
Word processing system/desktop publishing software	4 (8)	39 (76)		
General typeface of the main text, n (%)				
Serif[10]	**44 (85)**	13 (25)	**15.51 (5.51-48.98)**	< 0.0001, F
Sans-serif[11]	8 (15)	38 (75)		
General typeface of the main text headers, n (%)				
Serif[10]	**36 (69)**	4 (8)	**OR: 25.89 (7.68-116.13)**	< 0.0001, F
Sans-serif [11]	12 (23)	**44 (86)**	**OR: 0.05 (0.02-0.15)**	< 0.0001, F
Both	4 (8)	3 (6)	OR: 1.33 (0.21-9.56)	1.00, F
General typeface of the abstract text, n (%)	*N=52*	*N=12*	Not tested	
Serif[10]	19 (37)	**12 (100)**		
Sans-serif [11]	33 (63)	0		
General typeface of the abstract headers, n (%)			Not tested	
Serif[10]	11 (21)	9 (75)		
Sans-serif [11]	38 (73)	3 (25)		
No header in abstract	3 (6)	0		
Line spacing of the main document, n (%)			Not tested	
Single spaced	n/a	35 (69)		

	Journal (N=52)	Non-journal (N=51)	Odds Ratio (95% CI)	P-value, F/W[1]
>Single spaced	n/a	16 (31)		
Main document is presented in columns, n (%)			Not tested	
Single column	11 (21)	45 (88)		
Double column	39 (75)	3 (6)		
Triple column	2 (4)	0		
Other	0	3 (6)		
Graphic elements				
Use of typographic cues in the document, n (%)				
Bolded headers	52 (100)	51 (100)	OR not available	1.00, F
Bolded text, keywords or phrases	5 (10)	**17 (33)**	**0.22 (0.06-0.69)**	0.01, F
Use of colour text, keywords or phrases	12 (23)	16 (31)	0.66 (0.25-1.72)	0.38, F
User of underlining text, keywords or phrases	1 (2)	**29 (57)**	**0.02 (0.00-0.11)**	< 0.0001, F
Bullet lists	25 (48)	**44 (86)**	**0.15 (0.05-0.42)**	< 0.0001, F
Call out boxes	20 (38)	17 (33)	1.25 (0.52-3.04)	0.68, F
User of italics to highlight text, keywords or phrases	40 (77)	38 (75)	1.14 (0.42-3.11)	0.82, F
Main document text, n (%)				
Monochrome (black and white, or grey scale)	39 (75)	36 (71)	Monochrome/Colour	0.66, F
Colour	13 (25)	15 (29)	1.25 (0.48-3.28)	
Background colour				
white	13	15		
Text				
black	13	13		
dark blue	0	2		
Accent colour				
blue	7	8		
green	4	1		
teal	0	1		
purple	2	1		
mix	0	4		
Tables were presented in the main document, n (%)	45 (87)	45 (88)	0.86 (0.22-3.25)	1.00, F
Median, (IQR), [Range]	2 (3) [1-17]	6 (8.75) [1-33]		
Type of tables presented, n				
Characteristics of included studies	13	20	0.52 (0.20-1.30)	0.14, F
Outcome-specific data tables	6	**18**	**0.24 (0.07-0.72)**	0.01, F
Evidence tables (i.e., study level data tables)	18	20	0.82 (0.34-1.97)	0.69, F
General summary of findings tables	12	12	0.98 (0.35-2.70)	1.00, F
GRADE Summary of Findings tables	1	1	0.98 (0.01-78.43)	1.00, F
Quality assessment tables	5	4	1.20 (0.24-6.42)	1.00, F
Other (various e.g., non-study level for introduction)	29	56	Not tested	
Material provided in appendices, n (%)	27 (52)	**37 (73)**	**0.41 (0.17-1.00)**	0.04, F

	Journal (N=52)	Non-journal (N=51)	Odds Ratio (95% CI)	P-value, F/W[1]
Content of appendices:				
Search strategies/terms/sources searched	20	22		
Methods	1	4		
Inclusion criteria	2	1		
Data extraction forms	3	1		
List of included studies	1	1		
List of excluded studies	2	5		
Evidence tables (i.e., study level data)	10	19		
Table of study characteristics	5	11		
Outcome-specific data tables	0	3		
Quality assessment tool/evaluations	9	13		
GRADE Summary of Findings	4	2		
PRISMA Checklist	4	2		
Protocol	2	0		
Other (various material)	7	21		
Appendices not freely accessible (not able to assess)	1	0		
Figures were presented in the main document, n (%)	**38 (73)**	25 (49)	**2.79 (1.15-7.01)**	0.02, F
Median, (IQR), [Range]	1 (1) [1-8]	2 (3) [1-11]		
Types of figures presented, n				
PRISMA flow diagram	**34**	12	**6.02 (2.40-16.03)**	< 0.0001, F
Forest plot	1	1	0.98 (0.01-78.43)	1.00, F
Other (drawing or schematic etc.)	15	22	0.54 (0.22-1.30)	0.15, F
Funnel plot	0	0		
Figures included in appendices, n (%)	2 (4)	**21 (41)**	**0.06 (0.01-0.27)**	< 0.0001, F
Types of figures presented, n				
PRISMA flow diagram	0	15		
Forest plot	1	0		
Funnel plot	0	0		
Other (drawing etc.)	1	6		
Appendices not freely accessible (not able to assess)	1	0		
n/a (no figures included)	49	33		
Visual cues or institutional taglines used to brand the organization commissioning or producing the RR, n (%)			Not tested	
Institutional logos	4 (8)	52 (100)		
Header text or footer text	4 (8)	33 (65)		
Other (e.g., institutional symbol but not a specific logo)	2 (4)	0		
Other factors related to layout				
Placement of the methods section	*n=52*	*n=42*		
Front end of the document (e.g., for most journal publications)	**51 (98)**	21 (50)	**48.94 (7.01-2123.17)**	< 0.0001
Back end	0	4		

	Journal (N=52)	Non-journal (N=51)	Odds Ratio (95% CI)	P-value, F/W[1]
Other				
In the general text; no specific section	1 (2)	0		
Methods information in the appendices	0	17 (40)		
Placement of the 'key messages'	*n=3*	*n=24*		
Front end of the document	2	22	Not tested	
Back end of the document	1	2		
Other	0	0		
Placement of 'disclaimer'	*n=3*	*n=26*		
Front end of the document	2	25	Not tested	
Back end of the document	1	0		
Other	0	1		
Determination of the final report format, n (%)			Not tested	
Producer	n/a	1 (2)		
Commissioner	n/a	2 (4)		
Not reported/unclear	n/a	48 (94)		
Journal	52	n/a		
Stakeholder input with regards to the format and/or layout of the final end-product, n (%)			Not tested	
Yes	n/a	0		
No/Not Reported	n/a	51 (100)		
Additional materials listed as available upon request, n (%)	2 (4)	3 (6)	OR: 0.64 (0.05-5.87)	0.68, F
**Readability**				
SMOG Index, mean (SD)				
Abstract/Summary	13.91 (1.55)	14.24 (1.36)	MD (SE): -0.33 (0.29)	0.25, W
Introduction/Background	14.01 (1.91)	13.57 (1.55)	MD (SE): 0.44 (0.34)	0.20, W
Discussions/Conclusion	13.79 (1.68)	14.35 (2.29)	MD (SE): -0.56 (0.40)	0.16, W
Word count (cursory-level), mean (SD)[12] (entire document)	10,343 (10,051)	**17,393 (15,385)**	**MD (SE): -7,050 (2,566)**	**0.01, W**
Word count (cursory-level), mean (SD)[12] (main body of the text only)	6,708 (4,575)	**10,269 (8,818)**	**MD (SE): -3,561 (1,388)**	**0.01, W**

SD: standard deviation; MD: mean difference; SE: standard error; n/a: not applicable

1 F: Fisher's Exact Test for binomial counts; W: Welch's t-test for continuous score

2 IMRAD: a report format structured to include the following sections consecutively: Introduction, Methods, Results and Discussion sections of an original article

3 Graded entry: a report format organized to highlight decision-relevant, summarized information upfront with access to additional, more in-depth information.

4 1:3:25 format – a structure comprised of 1-page of main messages; 3-page executive summary; with an additional 25-pages allotted for the full report including references and appendices

5 SUPPORT Summary format – a structured designed to present the results of SRs to decision-makers with key messages from findings upfront, followed by context; search approach; search results; details of main findings including methodological quality of the evidence; applicability, equity, economic, monitoring and evaluation considerations; and references

6 Inverted pyramid format – a structure that emphasizes the conclusions or key messages upfront followed by brief (executive) summary, followed by a lengthier report that provides specific details for the reader. For the purposes of this study, this format similarly follows a 1:3:25 format but does not strictly adhere to this page count

7 Multicomponent – refers to a report with various components divided into 'chapters' or 'sections' beyond the typical IMRAD or general graded entry structures

[8] Excluding references and appendices
[9] 'Bannered' only refers to specifically labelled sections; if not labelled, this does not necessarily imply that the section or related content of a section was missing from the report(s); of the labels identified, some potentially overlap and could refer to similar concepts. However, content of the bannered sections was not formally assessed in this study.
[10] Serif font – category of typefaces that use small lines at the ends of characters (e.g., Times Roman, Courier)
[11] San-serif – category of typefaces that do not use small lines at the ends of characters (e.g., Arial, Calibri)
[12] Word count was calculated by converting PDF versions of the reports to Microsoft Word, and then using Word Count function
