# Supplementary material for: Assessing the format and content of journal published and non-journal published rapid review reports: A comparative study
Source: PLoS One. 2020 Aug 26;15(8):e0238025. doi: 10.1371/journal.pone.0238025 (PMC7449464; doi:10.1371/journal.pone.0238025)
Supplement: S2 Table — (PDF) [file pone.0238025.s010.pdf]

S8 Table. Peer Review Status and Salient Characteristics of Potential Predatory Journals

Unique Journals (n=47)	Publisher	Publication model	Peer Review	Salient characteristics of potential predatory journals												
				Scope Includes non-biomedical subjects alongside biomedical	Website spelling, grammar	Images: distorted/ fuzzy; deceiving or unauthorized	Homepage language targets authors	ICV promoted on website	Manuscript process description lacking	Email submission	Rapid publication	No retraction policy	No information, digital preservation of content	Very low article charge	Open access, retain or fail to note copyright	Email not professional
Appetite	Elsevier	Hyb	Y	×	×	×	×	×	×	×	×	×	×	×	×	×
Arch Dis Child	BMJ Publishing Group	Hyb	Y	×	×	×	×	×	×	×	×	×	×	×	×	×
Australian & New Zealand Journal of Psychiatry	SAGE	Hyb	Y	×	×	×	×	×	×	×	×	×	×	×	×	×
Australian Journal of Primary Health	CISRO Publishing	Hyb	Y	×	×	×	×	×	×	×	×	×	×	×	×	×
BMC Health Services Research	BMC	OA	Y	*Listed in the DOAJ*												
BMJ Open	BMJ Publishing Group	OA	Y	*Listed in the DOAJ*												
Canadian Journal of Gastroenterology and Hepatology	Hindawi Publishing Corp.	OA	Y	*Listed in the DOAJ*												
Canadian Journal of Occupational Therapy	SAGE	Hyb	Y	×	×	×	×	×	×	×	×	×	×	×	×	×
Canadian Respiratory Journal	Hindawi Publishing Corp.	OA	Y	*Listed in the DOAJ*												
Caries Res	Karger Publishers	Hyb	Y	×	×	×	×	×	×	×	×	✓	×	×	×	×
Child Abuse & Neglect	Elsevier	Hyb	Y	×	×	×	×	×	×	×	×	×	×	×	×	×
Clinical Neurology and Neurosurgery	Elsevier	Hyb	Y	×	×	×	×	×	×	×	×	×	×	×	×	×
Emergency Medicine Journal	BMJ Group	Hyb	Y	×	×	×	×	×	×	×	×	×	×	×	×	×
Eur J Nucl Med Mol Imaging	Springer	Hyb	Y	×	×	×	×	×	×	×	×	×	×	×	×	×
European Journal of Cardiovascular Nursing	SAGE	Hyb	Y	×	×	×	×	×	×	×	×	×	×	×	×	×
Gerontologist	Oxford Academic	Hyb	Y	×	×	×	×	×	×	×	×	×	×	×	×	×
Health Promotion International	Oxford Academic	Hyb	Y	×	×	×	×	×	×	×	×	×	×	×	×	×
Health Services and Delivery Research	National Institutes of Health Research (UK)	OA	Y	*Listed in the DOAJ*												
Health Technology Assessment	National Institutes of Health Research (UK)	OA	Y	*Listed in the DOAJ*												
Human Resources for Health	BMC (BioMed Central)	OA	Y	*Listed in the DOAJ*												
Infectious Diseases of Poverty	BMC (BioMed Central)	OA	Y	*Listed in the DOAJ*												
Int. J. Nurs. Educ. Scholarsh.	De Gruyter	Hyb	Y	×	×	×	×	×	×	×	×	✓	×	✓	×	×

Unique Journals (n=47)	Publisher	Publication model	Peer Review	Salient characteristics of potential predatory journals												
				Scope Includes non-biomedical subjects alongside biomedical	Website spelling, grammar	Images: distorted/ fuzzy; deceiving or unauthorized	Homepage language targets authors	ICV promoted on website	Manuscript process description lacking	Email submission	Rapid publication	No retraction policy	No information, digital preservation of content	Very low article charge	Open access, retain or fail to note copyright	Email not professional
Int. J. Oral Maxillofac. Surg.	Elsevier	Hyb	Y	✗	✗	✗	✗	✗	✗	✗	✗	✗	✗	✗	✗	✗
International Journal of Prisoner Health	Emerald Insight	Hyb	Y	✗	✗	✗	✗	✗	✗	✗	✗	✗	✗	✗	✗	✗
Journal of Emergency Medicine	Elsevier	Hyb	Y	✗	✗	✗	✗	✗	✗	✗	✗	✗	✗	✗	✗	✗
Journal of Head Trauma Rehabilitation	Wolters Kluwer Health	Hyb	Y	✗	✗	✗	✗	✗	✗	✗	✗	✓	✗	✗	✗	✗
Journal of the American Dental Association	Elsevier	Hyb	Y	✗	✗	✗	✗	✗	✗	✗	✗	✗	✗	✗	✗	✗
Journal of Advanced Nursing	Wiley	Hyb	Y	✗	✗	✗	✗	✗	✗	✗	✗	✗	✗	✗	✗	✗
Journal of Evidence-Based Medicine	Wiley	Hyb	Y	✗	✗	✗	✗	✗	✗	✗	✗	✗	✗	✗	✗	✗
Journal of Medical Screening	Sage	Hyb	Y	✗	✗	✗	✗	✗	✗	✗	✗	✗	✗	✗	✗	✗
Journal of Primary Care & Community Health	Sage	OA	Y	*Listed in the DOAJ*												
Journal of Public Health	Oxford Academic	Hyb	Y	✗	✗	✗	✗	✗	✗	✗	✗	✗	✗	✗	✗	✗
Journal of Research in Nursing	Sage	Hyb	Y	✗	✗	✗	✗	✗	✗	✗	✗	✗	✗	✗	✗	✗
Journal of Wound Care	MA Healthcare Limited, part of the Mark Allen Group of companies,	Subscript-ion	Y	✗	✗	✗	✗	✗	✗	✗	✗	✓	✗	✗	✗	✗
Nursing Ethics	SAGE	Hyb	Y	✗	✗	✗	✗	✗	✗	✗	✗	✗	✗	✗	✗	✗
Ontario Health Technology Assessment Series	Health Quality Ontario (HQO)	OA	N	✗	✗	✗	✗	✗	NA	NA	NA	✓	✓	NA	NA	✗
Perspectives in Public Health	SAGE	Hyb	Y	✗	✗	✗	✗	✗	✗	✗	✗	✗	✗	✗	✗	✗
PLOS Neglected Tropical Diseases	PLOS	OA	Y	*Listed in the DOAJ*												
PLoS ONE	PLOS	OA	Y	*Listed in the DOAJ*												
Public Health	Elsevier	Hyb	Y	✗	✗	✗	✗	✗	✗	✗	✗	✗	✗	✗	✗	✗
Rural and Remote Health	James Cook University	OA	Y	✗	✗	✗	✗	✗	✗	✗	✗	✗	✓	✗	✗	✗
Systematic Reviews	BMC – BioMed Centre	OA	Y	*Listed in the DOAJ*												
Teaching and Learning in Medicine	Taylor & Francis	Hyb	Y	✗	✗	✗	✗	✗	✗	✗	✗	✗	✗	✗	✗	✗
The Breast	Elsevier	Hyb	Y	✗	✗	✗	✗	✗	✗	✗	✗	✗	✗	✗	✗	✗
The Lancet	The Lancet	Hyb	Y	✗	✗	✗	✗	✗	✗	✗	✗	✗	✗	✗	✗	✗
Transpl Infect Dis	Wiley	Hyb	Y	✗	✗	✗	✗	✗	✗	✗	✗	✗	✗	✗	✗	✗

Unique Journals (n=47)	Publisher	Publication model	Peer Review	Salient characteristics of potential predatory journals												
				Scope Includes non-biomedical subjects alongside biomedical	Website spelling, grammar	Images: distorted/ fuzzy; deceiving or unauthorized	Homepage language targets authors	ICV promoted on website	Manuscript process description lacking	Email submission	Rapid publication	No retraction policy	No information, digital preservation of content	Very low article charge	Open access, retain or fail to note copyright	Email not professional
Tropic Journal of Pharmaceutical Research	Pharmacotherapy Group, Faculty of Pharmacy, University of Benin, Benin City, Nigeria	OA	Y	*Listed in the DOAJ*												

✗ - salient characteristic not identified; ✓ - salient characteristic identified; *DOAJ – Directory of Open Access Journals; Hyb – hybrid publishing model; IVC - The Index Copernicus Value; OA – open access; NA – not applicable*
